# Study of occlusal acoustic parameters in assessing masticatory performance

**DOI:** 10.1186/s12903-021-02018-9

**Published:** 2022-03-15

**Authors:** Yue Xia, Lu Wang

**Affiliations:** 1 Department of Orthodontics, Tianjin Stomatological Hospital, School of Medicine, Nankai University, 75 Dagu Road, Heping District, Tianjin, China; 2 Tianjin Key Laboratory of Oral and Maxillofacial Funcion Reconstruction, School of Medicine, Nankai University, 75 Dagu Road, Heping District, Tianjin, 300041 China

**Keywords:** Masticatory performance, Acoustic parameters, Kinematic parameters, D50 value, Bone-conduction tech

## Abstract

**Background:**

Previous masticatory studies have focused on a variety of measurements of foods and boluses or kinematic parameters and sound during mastication. To date, the masticatory sound research of has been limited due to the difficulties of sound collection and accurate analysis. Therefore, significant progress in masticatory sound has not been made. Meanwhile, the correlation between acoustic parameters and mastication performance remains unclear. For the purpose of exploring the acoustic parameters in measuring mastication performance, the bone-conduction techniques and sound analysis were used, and a statistical analysis of acoustic and occlusal parameters were conducted.

**Methods:**

The gnathosonic and chewing sounds of fifty-six volunteers with healthy dentate were recorded by a bone-conduction microphone and further analyzed by Praat 5.4.04 when intercuspally occluding natural foods (peanuts) were consumed. The granulometry of the expectorated boluses from the peanuts was characterized by the median particle size of the whole chewing sequence (D50^a^) and the median particle size during the fixed chewing strokes (D50^b^). The chewing time of the whole chewing sequence (CT^a^), the chewing time of the fixed chewing strokes (CT^b^), the chewing cycles (CC), and the chewing frequency (CF) were recorded and analyzed by the acoustic software. The acoustic parameters, including gnathosonic pitch, gnathosonic intensity, mastication sound pitch of the whole chewing sequence (MP^a^), mastication sound pitch of the fixed chewing strokes (MP^b^), mastication sound intensity of the whole chewing sequence (MI^a^) and mastication sound intensity of the fixed chewing strokes (MI^b^), were analyzed. Independent sample *t*-test, Spearman and Pearson correlation analyses were used where applicable.

**Results:**

Significant difference in parameters CC, MI^a^, CF and D50^a^ were found by sex (*t-*test, *p* < 0.01). The masticatory degree of the test foods was higher in women (CC, 24.25 ± 5.23; CF, 1.70 ± 0.21 s^−1^; D50^a^, 1655.07 ± 346.21 μm) than in men (CC, 18.14 ± 6.38; CF, 1.48 ± 0.18 s^−1^; D50^a^, 2159.21 ± 441.26 μm). In the whole chewing sequence study, a highly negative correlation was found between MI^a^ and D50^a^, and a highly positive correlation was found between MI^a^ and CF (r =  − 0.94, r = 0.82, respectively, *p* < 0.01). No significant correlation was found between the remaining acoustic parameters and mastication parameters. In the fixed chewing strokes study, a highly negative correlation was found between MI^b^ and D50^b^ (r =  − 0.85, *p* < 0.01). There was no significant correlation between the rest of the acoustic parameters and the mastication parameters.

**Conclusions:**

Mastication sound intensity may be a valuable indicator for assessing mastication. Acoustic analysis can provide a more convenient and quick method of assessing mastication performance.

**Supplementary Information:**

The online version contains supplementary material available at 10.1186/s12903-021-02018-9.

## Introduction

Restoring the ability to masticate food is one of the primary goals of dental treatment. Masticatory performance is defined as the ability to comminute test food [1]. In contrast to the early common method for assessing masticatory performance, which is a comminution method using a sieve [2], recent alternatives to the sieve method were introduced for assessing the particle size distribution. The main methods of assessing bolus particles include the digital scanning, spectrophotometer measurement of released dye and glucose released from fragmented test food particles [3–5]. In addition, the degree of mixing has been suggested as another way to assess mastication, which uses color-changeable chewing gum and two-color wax or gum as the test food [6–8]. Compared with mixing testing, the sieve method is probably better suited for research in recent studies [9, 10]. Recently, the method of bolus granulometry analysis, which is expressed as the D50 value, characterizes the test-food theoretical sieve size and the measurement of kinematic parameters before swallowing, which produces the bolus, has appeared to provide a good criterion for objectives with normal mastication [1, 11]. Therefore, bolus granulometry analysis combined with kinematic parameters may be an useful approach for further assessing masticatory performance.

Previous acoustic studies of occlusal sounds concentrated on gnathosonic and chewing sounds. Gnathosonic features the use of sounds generated as the teeth meet as an analogue of the quality of the occlusion [12]. The study of gnathosonic focused on occlusal interference and stability [13–16]. Other studies of chewing sound focused on the area of food texture and the relationship between mastication and swallowing [17–19]. However, the acoustic parameters were suggested to be merely reference indices during the observation of mastication behaviour, and there has been no further exploration of the relationship with masticatory performance in the previous studies [13–19]. Moreover, as a limitation of adapterization and analysis software in early research, it lacked research using complete sound capture and further analysis of acoustic parameters [20].

Based on the doubts about acoustic processing methods, the bone-conduction tech, which establishes an independent path of adapterization and provides a wider spectrum to annotate sound data, may be a good solution. In addition, the solid sound produced by occlusion could be collected purely through a bone-conducted microphone and could avoid rustling sounds in the air at the same time [21, 22]. Apart from improvements of the hardware, the new acoustic software provides a better way to calculate the sound frequencies, and the parameter of sound intensity were added during the experimental data processing which describes the loudness of the sound and directly reflects the energy of the soundwave for the sake of single data analysis in a previous study [23].

For the purpose of improving the study of occlusal sound, the bone-conduction tech was introduced to capture a more complete sound signal. Meanwhile, based on a previous study of occlusal sound, in which the acoustic parameter expressed a close connection with mastication, this study focused on the relationship between the masticatory performance and the occlusal sound signal. The possibility of evaluation of mastication by acoustic parameters was further explored. Additionally, we found that the acoustic signal could be captured and analyzed more completely and easily through measurements of the test-food bolus.

## Materials and methods

Fifty-six volunteers (28 men and 28 women) aged between 20 and 30 were recruited. All participants had complete natural dentition and had no functional mastication problems or dental restoration history. All participants had a normal overjet, overbite and Angle I molar relationship and could chew without unilateral mastication.

Every subject masticated 4 raw peanuts (weight 2.24 ± 0.16 g) from the start of chewing to the point before swallowing as naturally as possible, and the chewed boluses before swallowing were collected. Then, the subject chewed the same number of peanuts 21 times, which was the average time calculated from the former study, and the chewing sounds and bolus were collected as above.

A bone-conduction microphone from BONE VIBRATION HEADGEAR (Model: HG17BN-TX developed by TEMCo INDUSTRIAL LLC) [24, 25] linked to the recording device of SONY ZOOM H4n (Model: Dedicated Zoom AD-14 AC Adapter developed by Sony Group Corporation) was attached to the preauricular skin, which was used for recording the chewing sounds and gnathosonic (Additional file 1: Fig S. 3) [13–19]. Chewing sounds were captured by putting peanuts into the mouth to start chewing. Each gnathosonic was first recorded 10 times with mock chewing without any test food. All sound data were recorded in monochannel with an 8000 Hz sampling frequency and 16 bit sampling precision by the bone-conduction device.

After the collection of the acoustic parameters, the parameters were processed in Praat 5.4.04 (developed by Paul Boersma and David Weenink Phonetic Sciences, Division of Humanities, University of Amsterdam, Netherlands) [24, 25], to calculate the gnathosonic pitch (GP), gnathosonic intensity (GI), mastication sound pitch of the whole chewing sequence (MP^a^), mastication sound pitch of the fixed chewing strokes (MP^b^), mastication sound intensity of the whole chewing sequence (MI^a^) and the mastication sound intensity of the fixed chewing strokes (MI^b^).

Praat 5.4.04 was also used for the evaluation of kinematic parameters. The number of chewing cycles (CC), the chewing time of the whole chewing sequence (CT^a^) and the chewing time of fixed chewing strokes (CT^b^) were monitored and annotated accurately on the sound oscillogram by Praat. To calculate the chewing frequency (CF), CT^a^ was divided by CC in the statistics of each subject.

After rinsing with saliva through a 100-μm sieve in water, the chewed bolus was collected. After drying at 80 °C for 30 min, the bolus of each sample was dispersed on a transparent A4 acrylic sheet and then scanned to construct a 600 dpi image. With the analytical result from the images by Powdershape® (Model: Ringstrasse 29 CH-7324 Vilters developed by Innovative Sintering Technologies Ltd., Switzerland), the food bolus granulometry particle size and distribution were further evaluated in the manner of the median particle size value of the whole chewing sequence (D50^a^) and the median particle size value of fixed chewing strokes (D50^b^) which expressed the theoretical sieve size that would let through 50% of the particle weights. Thus, D50^a^ and D50^b^ decreased as the food boluses contained more small particles. According to a previous study, the two median particle size values of food boluses above 1 mm were recorded for each subject, and each natural food was averaged. As a result, a higher mastication degree produced smaller bolus granule particles.

The acoustic data collection phase of all subjects who wore the bone-conduction recording device was processed in a quiet single room. During the stage of gnathosonic collection, the subjects wore the bone-conduction device and were asked to bite naturally without test food 10 times into the intercuspal occlusion. Then, in the stage of chewing 4 raw peanuts, the chewing sounds were recorded and the bolus of the peanuts were collected. Finally, the sound and bolus of every subject, who masticated 4 raw peanuts with an average chewing stroke times (21 times) calculated from the former stage, were collected by the same method.

In the bolus processing phase, the masticated boluses of every participant were collected, rinsed and dried after chewing, and then processed by Powdershape®. The results of the bolus analysis were expressed in terms of the D50^a^ and D50^b^ values.

In the phase of the sound oscillogram data-processing by Praat, the periods of mastication were annotated on the timeline, and the values of CC, CT^a^, CT^b^, MI^a^, MI^b^, MP^a^, MP^b^, GI and GP were calculated by Praat. The result of CF was divided by CT and CC in the statistics of each subject.

Statistical analysis was performed using IBM SPSS®17 software. Independent *t*-test were conducted on the acoustic and masticatory parameters by gender. Statistical significance was set at *p* < 0.01. Spearman correlations were calculated between MP^a^, MP^b^ and the masticatory parameters (*p* < 0.01). Pearson correlations were calculated between the rest of the acoustic parameters and masticatory parameters (*p* < 0.01).

## Results

During the study of the entire chewing sequence, 56 volunteers (28 women, mean age 26.3 ± 1.4; 28 men, mean age 26.8 ± 2.3) met the clinical criteria for inclusion. Descriptive data of the masticatory sound, gnathosonic and mastication are shown in Table 1 and Additional file 1: Table S. 1.

**Table 1:** Age and mean values for acoustic parameters, kinematic parameters and D50^a^ (n = 56) for study of the entire chewing sequence

Parameters	Mean ± SD
Age, years	26.5 ± 1.7
D50^a^ (μm)	1907.14 ± 468.10
Kinematic parameters	
CC	21.20 ± 6.55
CT^a^ (s)	13.29 ± 3.75
CF(s^−1^)	1.59 ± 0.22
Acoustic parameters	
MI^a^ (dB)	61.00 ± 4.66
MP^a^ (Hz)	2234.30 ± 671.67
GI(dB)	54.76 ± 5.21
GP(Hz)	2906.50 ± 754.71

As seen in Table 2, Independent *t-*test indicated that no statistically significant differences in CT^a^, MP^a^, GP, or GI were found when comparing the parameters between different genders. There were statistically significant difference in CC, MI^a^, CF and D50^a^ by gender.

**Table 2:** Mean values (± SD) of D50^a^, GP, GI, MP^a^, MI^a^, CC, CT^a^ and CF of the whole chewing sequence with the quantitative food by gender

Parameters	Mean ± SD		Independent Samples *t* test Comparisons
	Male (n = 28)	Female (n = 28)	
D50^a^ (μm)	2159.21 ± 441.26	1655.07 ± 346.21	*p* < 0.01
CC	18.14 ± 6.38	24.25 ± 5.23	*p* < 0.01
CT^a^ (s)	12.33 ± 4.45	14.26 ± 2.63	NS*
CF (s^−1^)	1.48 ± 0.18	1.70 ± 0.21	*p* < 0.01
MI^a^ (dB)	57.64 ± 3.35	64.35 ± 3.11	*p* < 0.01
MP^a^ (Hz)	2234.92 ± 810.47	2233.68 ± 511.99	NS*
GI (dB)	54.99 ± 6.11	54.53 ± 4.23	NS*
GP (Hz)	3008.25 ± 861.84	2804.75 ± 629.30	NS*

*There was no statistically significant difference

**Fig. 1:**
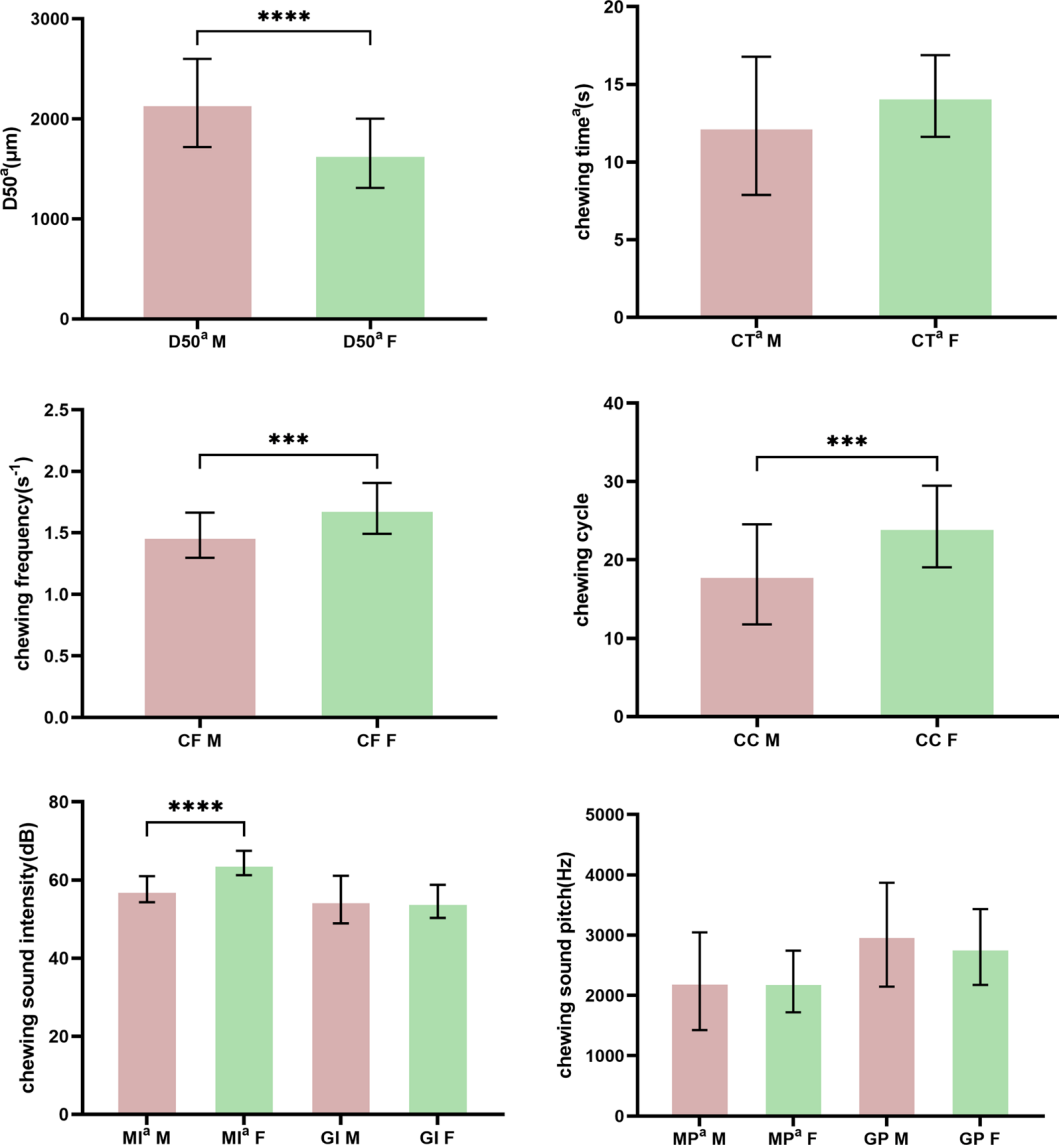
Mean values (± SD) of experimental data of the whole chewing sequence with the quantitative food compared between different genders. Intragroup comparisons were made with Independent *t*-test (***p < 0.001, ****p < 0.0001)

As shown in Fig. 1, the mean values (± SD) of men were significantly lower than those of women in the comparison of the kinematic parameters of the CC and CF. Meanwhile the mean values (± SD) of D50^a^ showed that the values of men were significantly higher than women. Moreover, in the statistical results of the acoustic parameters, MI^a^ approached compliance with kinematic parameters, and the value for females significantly exceeded that of males.

**Fig. 2:**
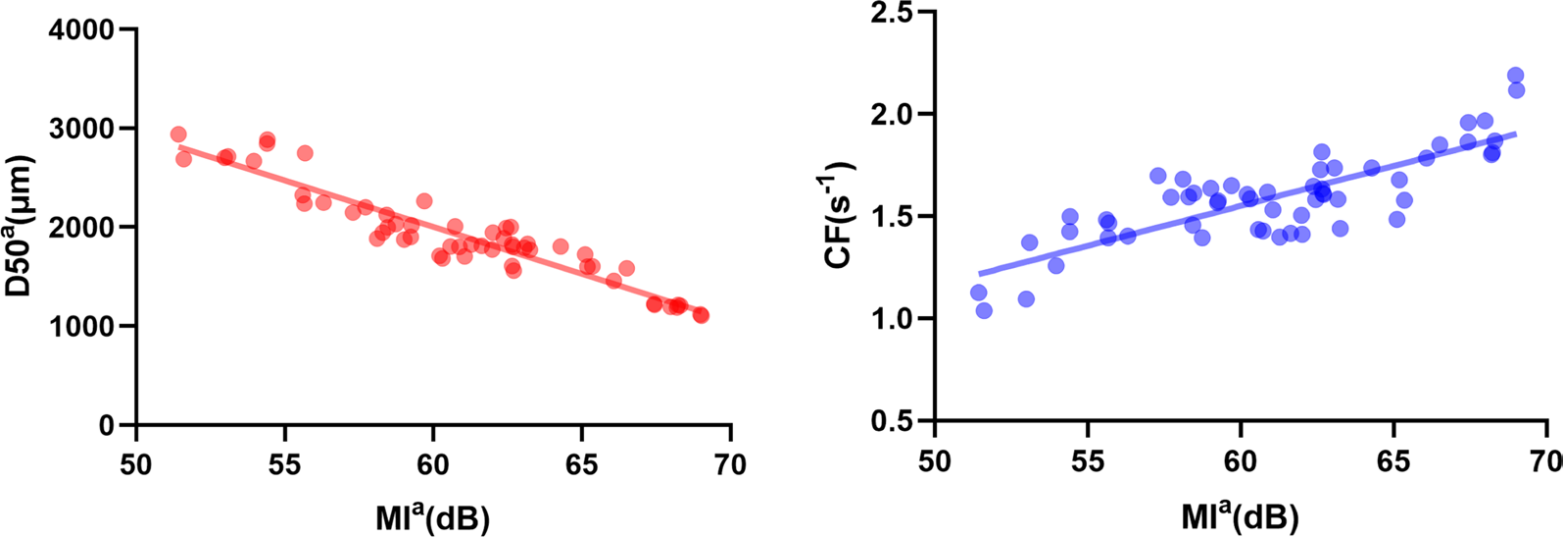
Scatter plot graph of MI^a^/D50^a^ and MI^a^/CF in the study of the whole chewing sequence with quantitative food

As in Table 3, Fig. 2 and Additional file 1: Fig. S. 1, Pearson’s correlation coefficients showed that MI^a^ was significantly negatively correlated with D50^a^ (r =  − 0.94), and was significantly positively correlated with CF (r = 0.82), which means that MI^a^ may be a sensitive indicator of masticatory kinematics and degree of mastication. Meanwhile MI^a^ and CC had a low correlation (r = 0.46), indicating that MI^a^ may not be correlated with CC. In the Spearman analysis and the rest of the Pearson’s analysis, there were no statistical correlation for any other acoustic parameters (MP^a^, GP or GI) or masticatory parameters (CC, CT^a^, CF or D50^a^).

**Table 3:** Pearson correlation analysis between acoustic parameters GP, GI, MI^a^ and masticatory parameters

	Pearson value			Spearman value
	GP (Hz)	GI (dB)	MI^a^ (dB)	MP^a^ (Hz)
D50^a^ (μm)	0.07	0.12	− 0.94*	− 0.09
CC	− 0.05	0.12	0.46	0.01
CT^a^ (s)	− 0.03	0.11	0.05	− 0.06
CF (s^−1^)	− 0.13	0.04	0.82*	0.26

Spearman correlation between MP^a^ and masticatory parameters

*r >0.80 or r < −0.80, highly correlated

During the fixed chewing strokes study, descriptive data of masticatory sound, gnathosonic and mastication with every volunteer chewed 4 raw peanuts 21 times are shown in Table 4 and Additional file 1: Table S. 2.

**Table 4:** Mean value parameter data of the quantitative test food (peanuts) with fixed chewing strokes (21 times)

Parameters	Mean ± SD
D50^b^ (μm)	1888.21 ± 203.08
CT^b^ (s)	13.16 ± 1.89
MI^b^ (dB)	59.20 ± 1.95
MP^b^ (Hz)	2155.44 ± 429.94
GI (dB)	54.76 ± 5.21
GP (Hz)	2906.50 ± 754.71

**Fig. 3:**
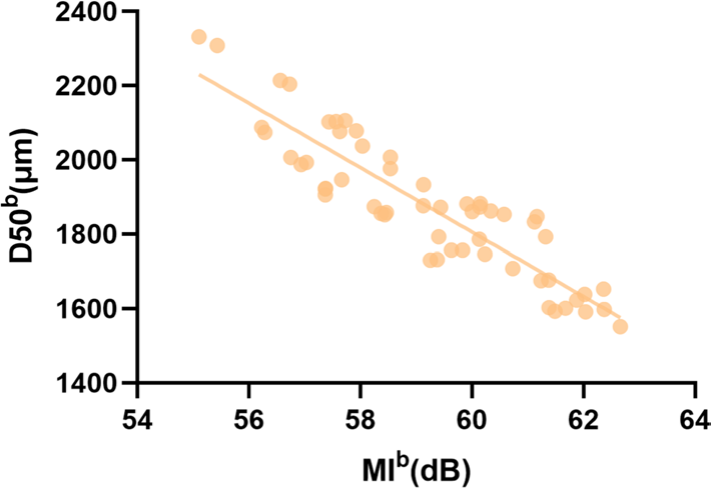
Scatter plots graph of MI^b^/D50^b^ of the quantitative test food (peanuts) with fixed chewing strokes (21 times)

As in Table 5, Fig. 3 and Additional file 1: Fig. S. 2, Pearson’s correlation coefficients showed that MI^b^ was significantly negatively correlated with D50^b^ (r =  − 0.85). In the Spearman analysis and the rest of the Pearson’s analysis, there was no statistical correlation for the other acoustic parameters (MP^b^, GP and GI) and masticatory parameters (CT^b^, D50^b^).

**Table 5:** Correlation analysis between the acoustic parameters and masticatory parameters of the quantitative test food (peanuts) with fixed chewing strokes (21 times)

	Pearson value			Spearman value
	GP (Hz)	GI (dB)	MI^b^ (dB)	MP^b^ (Hz)
D50^b^ (μm)	0.02	0.23	− 0.85*	0.07
CT^b^ (s)	− 0.09	0.18	− 0.15	0.09

*r >0.80 or r < −0.80, highly correlated

## Discussion

This study, for the first time, investigated the correlation between acoustic parameters and masticatory parameters to assess masticatory performance. The sound index, the mastication sound intensity (MI), showed a significant difference between genders in evaluating the masticatory performance, and it was significantly correlated with the critical masticatory parameters in two chewing studies. Moreover, compared to the masticatory parameters, the acoustic parameters showed more accuracy and comprehensiveness during data collection and more convenience in experimental process. This demonstrated that the further study of acoustic parameters evaluating mastication is meaningful.

Considering the sound sensitivity, the natural test food, raw peanut, was chosen to act as the masticatory sample, which is more fragile than many other test samples [26]. Compared with CC and CT, which were demonstrated to be valid indicators that can be used as alternatives to the homologous indicators obtained by electromyography in early research [27], CF was more valuable than the other parameters [28–32]. Meanwhile, analysis of the indicators of the median particle size value (D50) of the food bolus granulometry images was a more reliable and effective method to assess the masticatory performance [9–11]. Concomitant evaluations of bolus granulometry and kinematic parameters in the same mastication process appeared to be good criteria for assessing masticatory performance [33]. In summary, based on a previous study, the median particle size (D50) was a better indicator to evaluate the mastication in various research, and kinematic parameters provided valuable guidance [28, 33–38].

In the analysis of difference by sex, CC, CF, D50^a^ and MI ^a^ showed significant statistical differences. In a previous study, on the condition that subjects were asked to chew the given food, the masticatory motion intensity of women was more strenuous than that of men, and the indicator of chewing frequency (CF) and the median particle size (D50) presented the significant differences between men and women [28, 31, 39, 40]. These results are in accordance with this study, and the variation in CC, CF and D50^a^ in this study indicated that women showed a higher chewing degree while masticating the quantitative food [11, 26, 27, 35]. Meanwhile, in this study, the analysis of MI^a^, which represents the loudness and energy of the sound wave [23], displayed a significant difference by sex. As the sound wave created by mastication was a single tone and involved solid-bone transmission, the parameter of mastication sound intensity (MI) indicated the sound energy of the occlusion produced from masticatory movement [41, 42]. Hence, the variation in MI^a^ by sex was in accordance with D50^a^, CF and CC. This result suggested that an indicator of mastication sound intensity (MI) would be valuable in evaluating mastication.

In the following analysis of correlation among acoustic and masticatory parameters, MI^a^ revealed that a significant negative correlation existed with D50^a^ (r =  − 0.94), and a significant positive correlation existed with CF (r = 0.82). In the studies of the masticatory performance with fixed chewing strokes, the test food was in accordance with a previous study, and the number of chewing strokes was the average chewing cycle [43]. As shown in the results, in all acoustic parameters, MI^b^ was significantly negatively correlated with D50^b^ (r =  − 0.85). As a result, the more strenuous the mastication subjects made, the more energetic the soundwaves and the smaller food the bolus they produced. These results indicated that mastication sound intensity (MI) would be a meaningful indicator of masticatory performance.

Early studies mainly focused on the gnathosonic which is related to occlusal stability and interference [13–16]. To date, the related studies have mainly been based on the classification in occlusal sounds of Watt’s work [44, 45]. Due to limitations of adapterization and analysis methods, the subsequent doubt centralized the methodology, which lacked meaningful data analysis and complete sound capture [20]. Hence, there were few reports about acoustic studies in the stomatology area. The problem was due to the narrow acoustic range of occlusal sound and the deficiency of noise filtering in conventional sound sensors. With the bone-conducted tech which established the independent skeleton path of sound transmission and provided a wider spectrum to annotate the sound data, sound capture could be accomplished without the disturbance of background noise [21, 22].

In the vast majority of studies about gnathosonic and chewing sounds, the indicator analysis was confined to sound frequency and was compared with electromyography. Although the sound frequency was significantly correlated with the value of electromyography, it could not be used to evaluate the mastication process [13–19, 41]. Moreover, previous studies of chewing sound focused on the behavior of chewing and swallowing and the measurement of food texture, rather than investigating masticatory performance [16–18, 42]. Essentially, the sound frequency is more relevant to the vibrational speed of soundwaves than to the vibrational energy [31]. It is easy to understand that the mastication sound pitch (MP) and gnathosonic pitch (GP) were not correlated with the masticatory parameters. For gnathosonic intensity (GI), it was concluded that the physiological status of normal mastication varies significantly from that of occlusion without food. Furthermore, the gnathosonic intensity (GI) represents the energy of the direct contact of molars, which may be related to the texture of surface of molars and method of occlusion, instead of the status of mastication. In summary, the mastication sound intensity (MI) could be a meaningful indicator to evaluate the masticatory performance.

In addition, we found that, compared with conventional methods, the acoustic parameter data were acquired more accurately and conveniently by annotating the waveform in sound analysis software. The chewing cycle (CC) could be counted synchronously by the emergence of soundwave crests. Meanwhile the chewing time (CT) could be annotated distinctly with the variation of the chewing sound waveform. Furthermore, we could accomplish all of these experiments in a single quiet room without laboratory processing. In summary, the bone-conduction equipment and sound analysis has potential for masticatory studies.

## Conclusions

In this research, bone-conduction equipment was used and the occlusal sound signal was further analyzed to study the correlation between acoustic parameters and masticatory parameters. The indicator of mastication sound intensity (MI) was found to have a strong correlation with the state of the food bolus (D50) and chewing frequency (CF) in the entire chewing sequence study. In the study of fixed chewing strokes, the indicator of mastication sound intensity (MI) was highly correlated with the food bolus (D50). The results indicated that the indicator of mastication sound intensity (MI) might be valuable in assessing masticatory performance. The highlight of this research was the application of a bone-conduction tech, which improved the integrity of sound capture and the precision of sound analysis. Furthermore, compared to the complex laboratory procedures of handling food boluses, the measurement of occlusal sounds could be completed by researchers in any quiet places. However, this study merely concentrated on the occlusion of peanuts. Further studies of the occlusal sounds are ongoing to expand the varieties of the test foods and to explore the diverse characteristics of acoustic parameters in different chewing phases. Additionally, we are preparing to establish a masticatory acoustics database of normal people, and to evaluate the mastication of people in different age periods and patients after the dental restoration and treatment of temporomandibular disorder.

## Supplementary Information

**Additional file 1: Table S1.** Acoustic and masticatory parameters data of the whole chewing sequence and acoustic parameters data of gnathosonic. **Table S2.** Acoustic parameters and masticatorty parameters data of quantitative test food (peanuts) with fixed chewing strokes (21 times). **Figure S1.** The scatter plots graph of the acoustic and masticatory parameters in the whole chewing sequence study. **Figure S2.** The scatter plots graph of MI^b^, MP^b^, D50^b^ and CT^b^ of in the fixed chewing strokes study (21 times). **Figure S3.** The bone conduction microphone and Sony record device used in this study.

## Data Availability

All data generated or analysed during this study are included in this published article [and its supplementary information files].

## Abbreviations

D50: Median particle size
CT: Chewing time
CC: Chewing cycles
CF: Chewing frequency
GP: Gnathosonic pitch
GI: Gnathosonic intensity
MP: Mastication sound pitch
MI: Mastication sound intensity
