# Supplementary material for: Study of occlusal acoustic parameters in assessing masticatory performance
Source: BMC Oral Health. 2022 Mar 15;22:74. doi: 10.1186/s12903-021-02018-9 (PMC8925045; doi:10.1186/s12903-021-02018-9)
Supplement: Supplementary file 1 — Additional file 1: Table S1. Acoustic and masticatory parameters data of the whole chewing sequence and acoustic parameters data of gnathosonic. Table S2. Acoustic parameters and masticatorty parameters data of quantitative test food (peanuts) with fixed chewing strokes (21 times). Figure S1. The scatter plots graph of the acoustic and masticatory parameters in the whole chewing sequence study. Figure S2. The scatter plots graph of MIb, MPb, D50b and CTb of in the fixed chewing strokes study (21 times). Figure S3. The bone conduction microphone and Sony record device used in this study. [file 12903_2021_2018_MOESM1_ESM.doc]

Supporting Information

**Study of Occlusal Acoustic Parameters in Assessing Masticatory Performance**

Content

**Table S. 1.** Acoustic and masticatory parameters data of the whole chewing sequence and acoustic parameters data of gnathosonic ....….……. ……. ……. ……. ………….2

**Table S. 2.** Acoustic parameters and masticatorty parameters data of quantitative test food (peanuts) with fixed chewing strokes (21 times)…………………………...…. .…4

**Figure S. 1.** The scatter plots graph of the acoustic and masticatory parameters in the whole chewing sequence study..................................................................................………6

**Figure S. 2.** The scatter plots graph of MI^b^, MP^b^, D50^b^ and CT^b^ of in the fixed chewing strokes study (21times) ………..……………...…………………………………………….…8

**Figure S. 3.** The bone-conduction microphone and Sony record device...………………………9

**Table S1.** Acoustic and masticatory parameters data of the whole chewing sequence and acoustic parameters data of gnathosonic

| Sex | MP^a^ (Hz) | MI^a^ (dB) | CT^a^ (s) | CC | CF (s^-1^) | D50^a^ (μm) | GP (Hz) | GI (dB) |
| --- | --- | --- | --- | --- | --- | --- | --- | --- |
| male | 2000.20 | 60.21 | 17.66 | 28 | 1.61 | 1712 | 2793.48 | 66.15 |
| male | 1894.32 | 60.30 | 17.03 | 27 | 1.59 | 1686 | 2794.77 | 57.25 |
| male | 1758.30 | 61.05 | 9.84 | 18 | 1.53 | 1704 | 4115.01 | 55.95 |
| male | 1808.84 | 62.71 | 7.87 | 15 | 1.60 | 1563 | 2489.57 | 51.26 |
| male | 2017.67 | 57.30 | 14.13 | 24 | 1.70 | 2148 | 2801.47 | 62.22 |
| male | 1793.66 | 58.74 | 14.35 | 20 | 1.39 | 2033 | 3236.62 | 46.57 |
| male | 3103.84 | 53.96 | 10.40 | 11 | 1.26 | 2667 | 1703.22 | 62.30 |
| male | 2229.96 | 60.58 | 10.49 | 12 | 1.43 | 1806 | 2281.41 | 50.34 |
| male | 2013.61 | 56.31 | 7.13 | 10 | 1.40 | 2249 | 4544.40 | 55.74 |
| male | 2090.15 | 55.66 | 6.46 | 9 | 1.40 | 2238 | 4860.91 | 50.25 |
| male | 2568.79 | 58.46 | 6.20 | 10 | 1.61 | 1997 | 2744.59 | 47.74 |
| male | 2180.30 | 58.11 | 7.73 | 13 | 1.68 | 1884 | 3581.87 | 61.32 |
| male | 2031.82 | 59.02 | 10.39 | 17 | 1.64 | 1876 | 2232.87 | 44.56 |
| male | 1966.31 | 60.88 | 9.27 | 15 | 1.62 | 1803 | 2693.45 | 54.62 |
| male | 1877.95 | 58.29 | 10.03 | 16 | 1.60 | 1945 | 2657.33 | 62.07 |
| male | 1837.23 | 62.65 | 6.06 | 11 | 1.81 | 1609 | 3032.20 | 59.61 |
| male | 1658.90 | 53.09 | 11.68 | 16 | 1.37 | 2716 | 2855.12 | 57.98 |
| male | 2061.40 | 55.69 | 9.56 | 14 | 1.47 | 2748 | 2423.16 | 63.20 |
| male | 4683.76 | 51.43 | 15.10 | 17 | 1.13 | 2938 | 2561.48 | 52.54 |
| male | 5110.57 | 54.42 | 11.36 | 17 | 1.50 | 2886 | 2537.17 | 51.84 |
| male | 1837.80 | 54.41 | 11.94 | 17 | 1.42 | 2845 | 1886.23 | 46.70 |
| male | 1926.08 | 59.24 | 14.05 | 22 | 1.57 | 1901 | 4653.74 | 53.28 |
| male | 1951.47 | 55.61 | 18.80 | 32 | 1.48 | 2324 | 4832.15 | 55.17 |
| male | 2547.78 | 57.72 | 17.85 | 32 | 1.59 | 2203 | 2398.12 | 60.27 |
| male | 1777.72 | 51.61 | 21.20 | 22 | 1.04 | 2689 | 2960.44 | 62.24 |
| male | 2153.86 | 52.99 | 21.94 | 24 | 1.09 | 2701 | 3429.11 | 54.11 |
| male | 1858.84 | 61.99 | 13.30 | 20 | 1.50 | 1776 | 2337.46 | 48.26 |
| male | 1836.59 | 61.63 | 13.43 | 19 | 1.42 | 1811 | 2793.69 | 46.19 |
| female | 2545.43 | 67.42 | 15.03 | 28 | 1.86 | 1224 | 2645.38 | 57.86 |
| female | 2046.65 | 68.20 | 15.55 | 28 | 1.80 | 1187 | 3007.43 | 53.13 |
| female | 2077.51 | 62.65 | 12.25 | 20 | 1.63 | 1826 | 2596.96 | 49.23 |
| female | 3303.76 | 61.28 | 15.02 | 21 | 1.40 | 1824 | 2746.15 | 47.84 |
| female | 1920.55 | 66.51 | 18.39 | 34 | 1.85 | 1586 | 3651.24 | 52.66 |
| female | 2009.13 | 66.07 | 17.93 | 32 | 1.79 | 1458 | 1997.26 | 51.25 |
| female | 2068.27 | 65.35 | 10.78 | 17 | 1.58 | 1606 | 2865.53 | 52.10 |
| female | 2812.45 | 65.19 | 10.73 | 18 | 1.68 | 1603 | 4467.40 | 54.11 |
| female | 1968.27 | 58.43 | 17.18 | 25 | 1.46 | 2124 | 4708.26 | 51.99 |
| female | 1526.71 | 59.27 | 17.14 | 27 | 1.58 | 2018 | 3122.08 | 56.56 |
| female | 1976.49 | 65.11 | 14.15 | 21 | 1.48 | 1726 | 2904.45 | 55.59 |
| female | 2687.45 | 64.28 | 10.37 | 18 | 1.74 | 1805 | 2862.16 | 53.66 |
| female | 1739.59 | 63.17 | 18.95 | 30 | 1.58 | 1833 | 1904.52 | 62.74 |
| female | 1959.02 | 63.25 | 18.77 | 27 | 1.44 | 1772 | 2513.65 | 54.00 |
| female | 2433.78 | 68.31 | 13.93 | 26 | 1.87 | 1209 | 2338.47 | 49.23 |
| female | 1862.92 | 68.23 | 13.26 | 24 | 1.81 | 1216 | 2043.87 | 46.21 |
| female | 3658.37 | 68.99 | 13.71 | 30 | 2.19 | 1117 | 2472.17 | 55.51 |
| female | 2846.44 | 69.02 | 15.13 | 32 | 2.12 | 1104 | 3160.26 | 54.26 |
| female | 2116.55 | 63.06 | 11.53 | 20 | 1.74 | 1793 | 2684.53 | 52.52 |
| female | 1811.77 | 62.68 | 13.04 | 21 | 1.61 | 1802 | 2896.45 | 58.04 |
| female | 1544.95 | 62.01 | 12.76 | 18 | 1.41 | 1944 | 2746.51 | 57.02 |
| female | 1897.49 | 62.45 | 14.48 | 20 | 1.58 | 1992 | 2551.08 | 56.31 |
| female | 2521.56 | 62.37 | 9.22 | 17 | 1.65 | 1889 | 2094.66 | 63.90 |
| female | 1717.49 | 62.61 | 11.42 | 22 | 1.73 | 2003 | 2617.39 | 59.02 |
| female | 1895.43 | 60.73 | 13.31 | 19 | 1.43 | 2007 | 3009.26 | 50.41 |
| female | 2498.14 | 59.70 | 14.55 | 24 | 1.65 | 2265 | 2761.69 | 56.18 |
| female | 2617.74 | 67.98 | 14.75 | 29 | 1.97 | 1194 | 2536.21 | 54.32 |
| female | 2478.99 | 67.44 | 15.83 | 31 | 1.96 | 1215 | 2627.91 | 61.21 |

**Table S2.** Acoustic parameters and masticatorty parameters data of quantitative test food (peanuts) with fixed chewing strokes (21 times)

| Num | MP^b^ (Hz) | MI^b^ (dB) | CT^b^ (s) | D50^b^ (μm) | GP (Hz) | GI (dB) |
| --- | --- | --- | --- | --- | --- | --- |
| 1 | 1954.26 | 59.63 | 15.24 | 1757 | 2793.48 | 66.15 |
| 2 | 1753.12 | 62.66 | 13.47 | 1551 | 2794.77 | 57.25 |
| 3 | 1802.35 | 59.40 | 11.37 | 1793 | 4115.01 | 55.95 |
| 4 | 1834.28 | 61.67 | 12.26 | 1601 | 2489.57 | 51.26 |
| 5 | 1953.66 | 59.38 | 12.75 | 1732 | 2801.47 | 62.22 |
| 6 | 1802.14 | 61.12 | 13.38 | 1833 | 3236.62 | 46.57 |
| 7 | 2625.25 | 56.75 | 14.42 | 2006 | 1703.22 | 62.30 |
| 8 | 2154.36 | 62.36 | 15.05 | 1653 | 2281.41 | 50.34 |
| 9 | 2253.55 | 60.73 | 9.54 | 1708 | 4544.40 | 55.74 |
| 10 | 1996.92 | 59.83 | 10.66 | 1757 | 4860.91 | 50.25 |
| 11 | 2351.69 | 61.38 | 9.38 | 1603 | 2744.59 | 47.74 |
| 12 | 2003.82 | 58.43 | 12.26 | 1852 | 3581.87 | 61.32 |
| 13 | 1944.63 | 60.15 | 13.74 | 1882 | 2232.87 | 44.56 |
| 14 | 2153.53 | 61.16 | 11.14 | 1847 | 2693.45 | 54.62 |
| 15 | 1946.66 | 59.25 | 9.76 | 1730 | 2657.33 | 62.07 |
| 16 | 1826.03 | 61.49 | 14.70 | 1593 | 3032.20 | 59.61 |
| 17 | 1744.15 | 58.25 | 13.33 | 1874 | 2855.12 | 57.98 |
| 18 | 2058.33 | 57.37 | 11.12 | 1905 | 2423.16 | 63.20 |
| 19 | 3261.92 | 56.94 | 14.26 | 1987 | 2561.48 | 52.54 |
| 20 | 4018.43 | 58.46 | 13.37 | 1858 | 2537.17 | 51.84 |
| 21 | 2468.63 | 57.38 | 12.25 | 1923 | 1886.23 | 46.70 |
| 22 | 2410.11 | 59.91 | 10.67 | 1881 | 4653.74 | 53.28 |
| 23 | 1982.53 | 57.37 | 16.66 | 1921 | 4832.15 | 55.17 |
| 24 | 2231.97 | 61.37 | 15.53 | 1677 | 2398.12 | 60.27 |
| 25 | 1862.74 | 56.29 | 14.46 | 2074 | 2960.44 | 62.24 |
| 26 | 2007.01 | 57.67 | 13.61 | 1946 | 3429.11 | 54.11 |
| 27 | 1693.75 | 62.02 | 11.21 | 1639 | 2337.46 | 48.26 |
| 28 | 1906.51 | 62.37 | 12.26 | 1598 | 2793.69 | 46.19 |
| 29 | 2367.56 | 60.23 | 11.10 | 1746 | 2645.38 | 57.86 |
| 30 | 2157.34 | 58.37 | 14.53 | 1855 | 3007.43 | 53.13 |
| 31 | 2463.01 | 59.12 | 14.24 | 1876 | 2596.96 | 49.23 |
| 32 | 2994.59 | 57.44 | 13.31 | 2102 | 2746.15 | 47.84 |
| 33 | 1877.62 | 61.32 | 12.26 | 1793 | 3651.24 | 52.66 |
| 34 | 2104.27 | 60.00 | 11.37 | 1860 | 1997.26 | 51.25 |
| 35 | 2341.30 | 59.13 | 12.58 | 1933 | 2865.53 | 52.10 |
| 36 | 2574.36 | 57.73 | 15.64 | 2105 | 4467.40 | 54.11 |
| 37 | 2016.31 | 56.23 | 15.50 | 2087 | 4708.26 | 51.99 |
| 38 | 1657.40 | 55.11 | 14.96 | 2331 | 3122.08 | 56.56 |
| 39 | 2058.96 | 59.43 | 10.08 | 1872 | 2904.45 | 55.59 |
| 40 | 2805.39 | 57.93 | 12.96 | 2078 | 2862.16 | 53.66 |
| 41 | 1903.26 | 58.54 | 15.26 | 1976 | 1904.52 | 62.74 |
| 42 | 2046.81 | 60.14 | 14.18 | 1873 | 2513.65 | 54.00 |
| 43 | 2137.76 | 62.04 | 13.57 | 1591 | 2338.47 | 49.23 |
| 44 | 1933.58 | 61.88 | 13.38 | 1623 | 2043.87 | 46.21 |
| 45 | 3104.29 | 60.13 | 14.08 | 1786 | 2472.17 | 55.51 |
| 46 | 2530.60 | 61.24 | 14.33 | 1675 | 3160.26 | 54.26 |
| 47 | 1982.77 | 57.03 | 14.53 | 1993 | 2684.53 | 52.52 |
| 48 | 1903.25 | 58.54 | 13.53 | 2007 | 2896.45 | 58.04 |
| 49 | 1662.79 | 57.63 | 9.76 | 2076 | 2746.51 | 57.02 |
| 50 | 1953.03 | 56.73 | 12.15 | 2204 | 2551.08 | 56.31 |
| 51 | 2034.63 | 57.56 | 15.53 | 2103 | 2094.66 | 63.90 |
| 52 | 1902.75 | 58.04 | 12.26 | 2037 | 2617.39 | 59.02 |
| 53 | 1804.25 | 55.43 | 11.53 | 2308 | 3009.26 | 50.41 |
| 54 | 2184.90 | 56.56 | 13.47 | 2214 | 2761.69 | 56.18 |
| 55 | 2005.63 | 60.34 | 16.69 | 1862 | 2536.21 | 54.32 |
| 56 | 2194.14 | 60.57 | 16.54 | 1853 | 2627.91 | 61.21 |

**
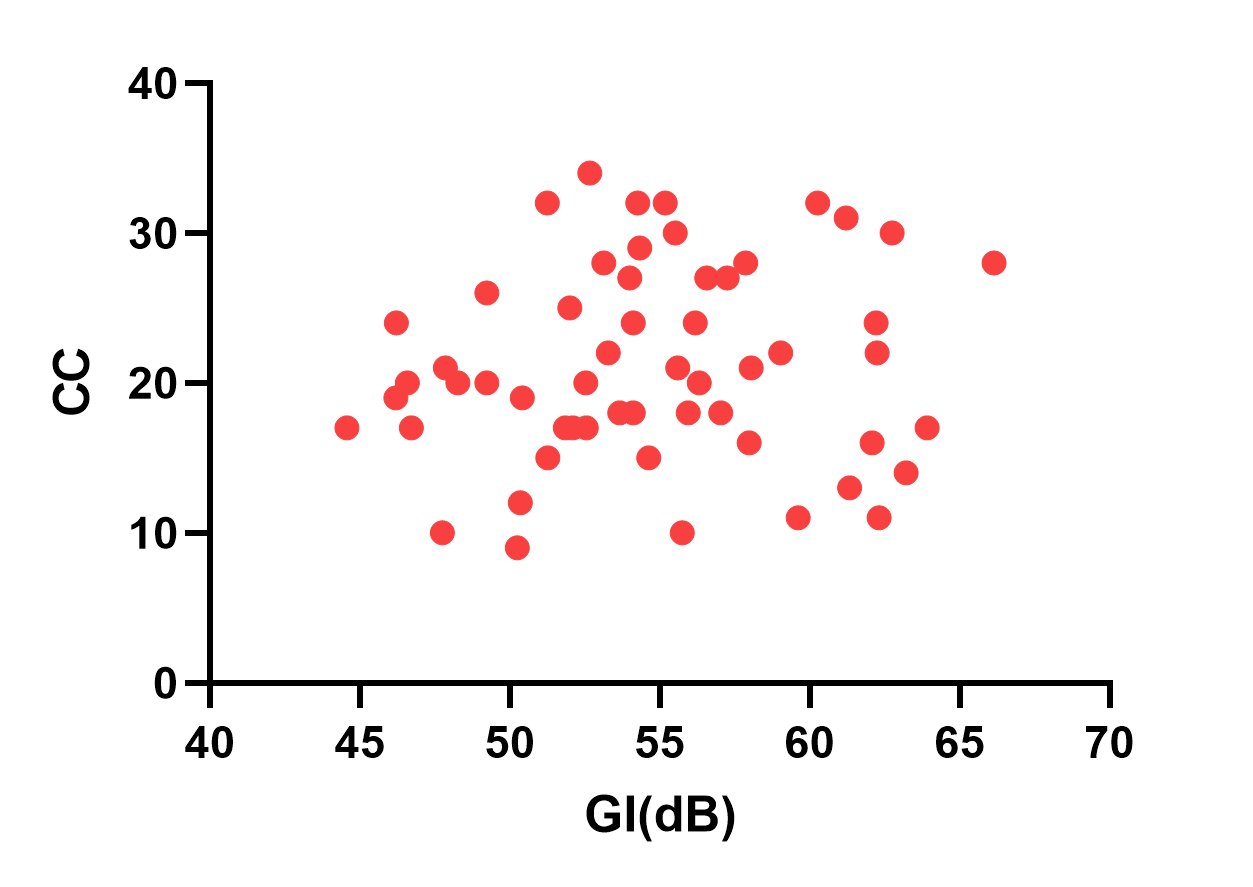

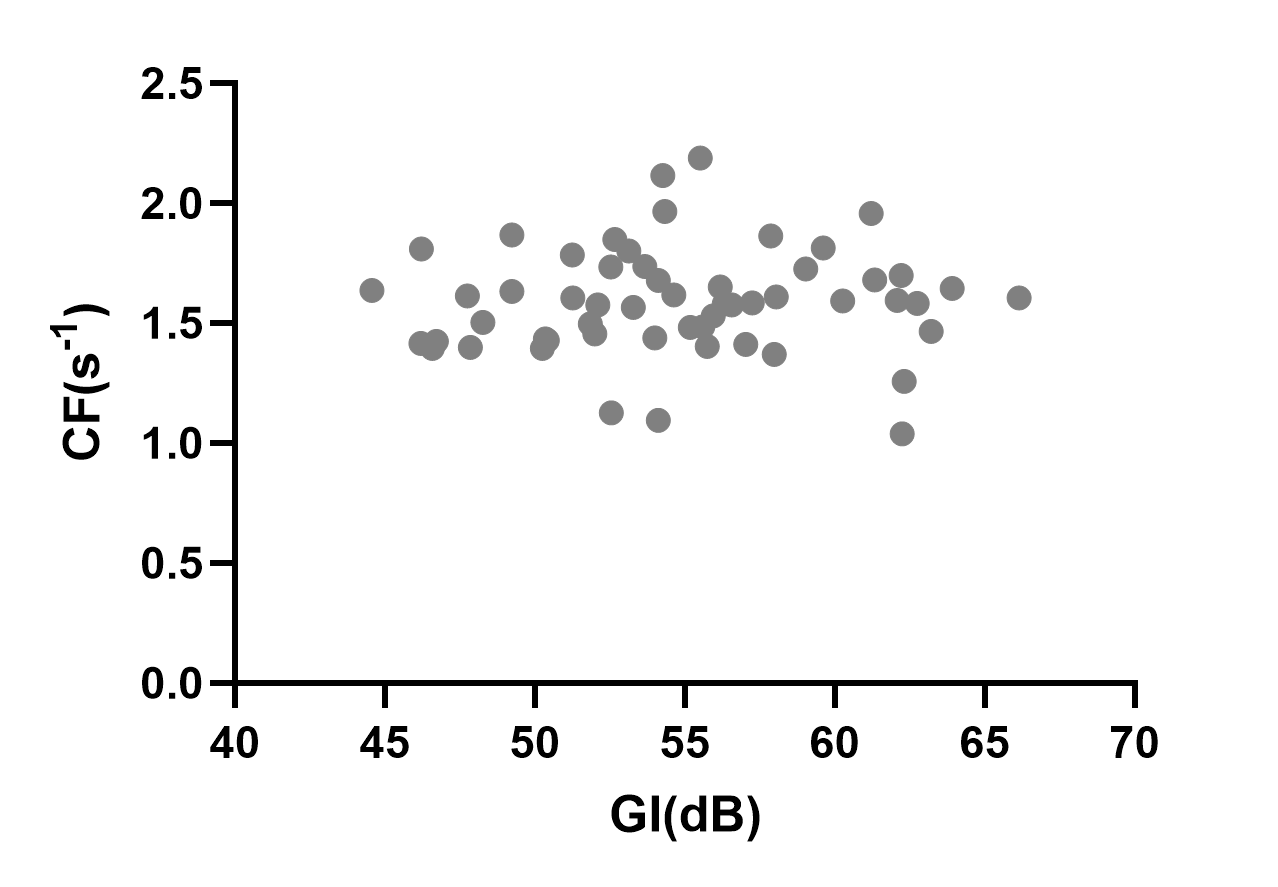

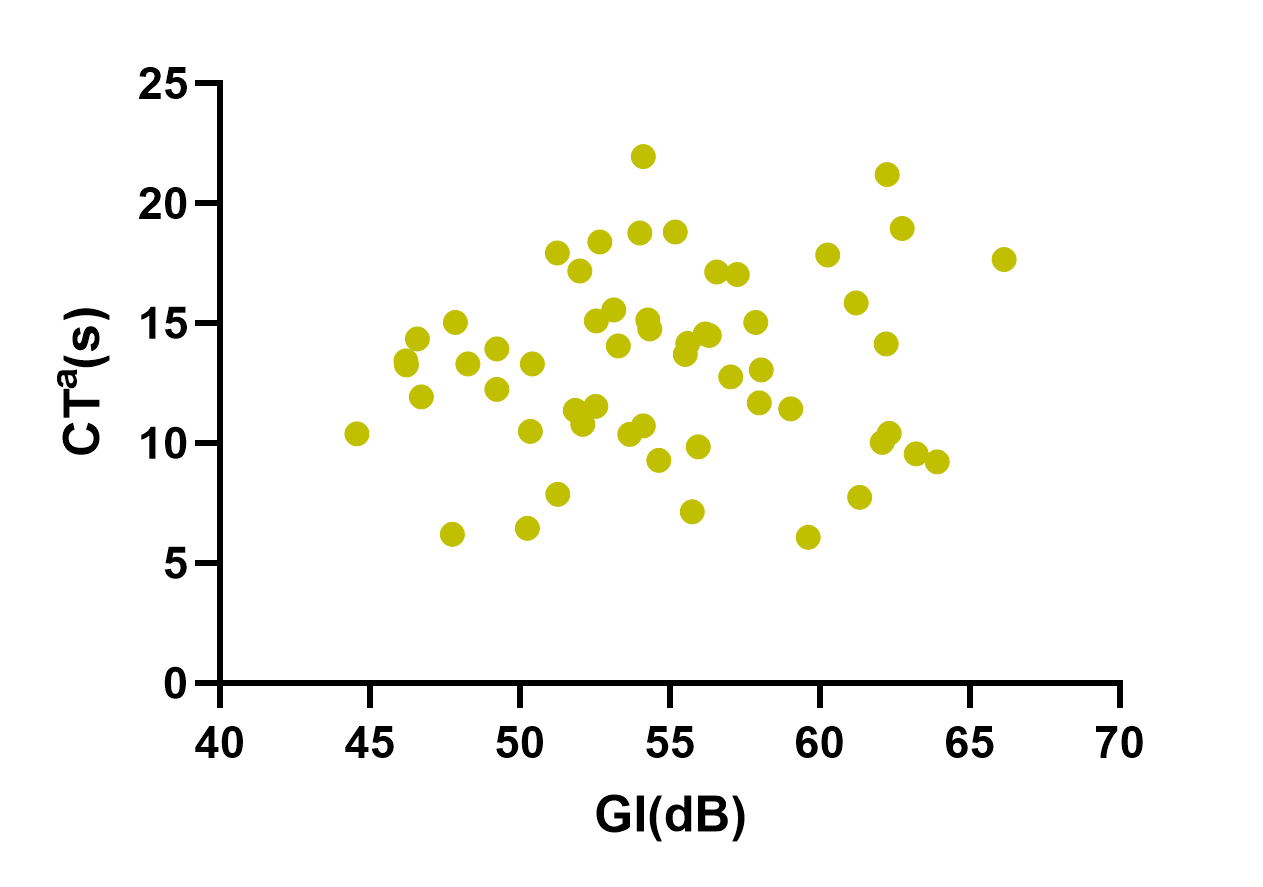

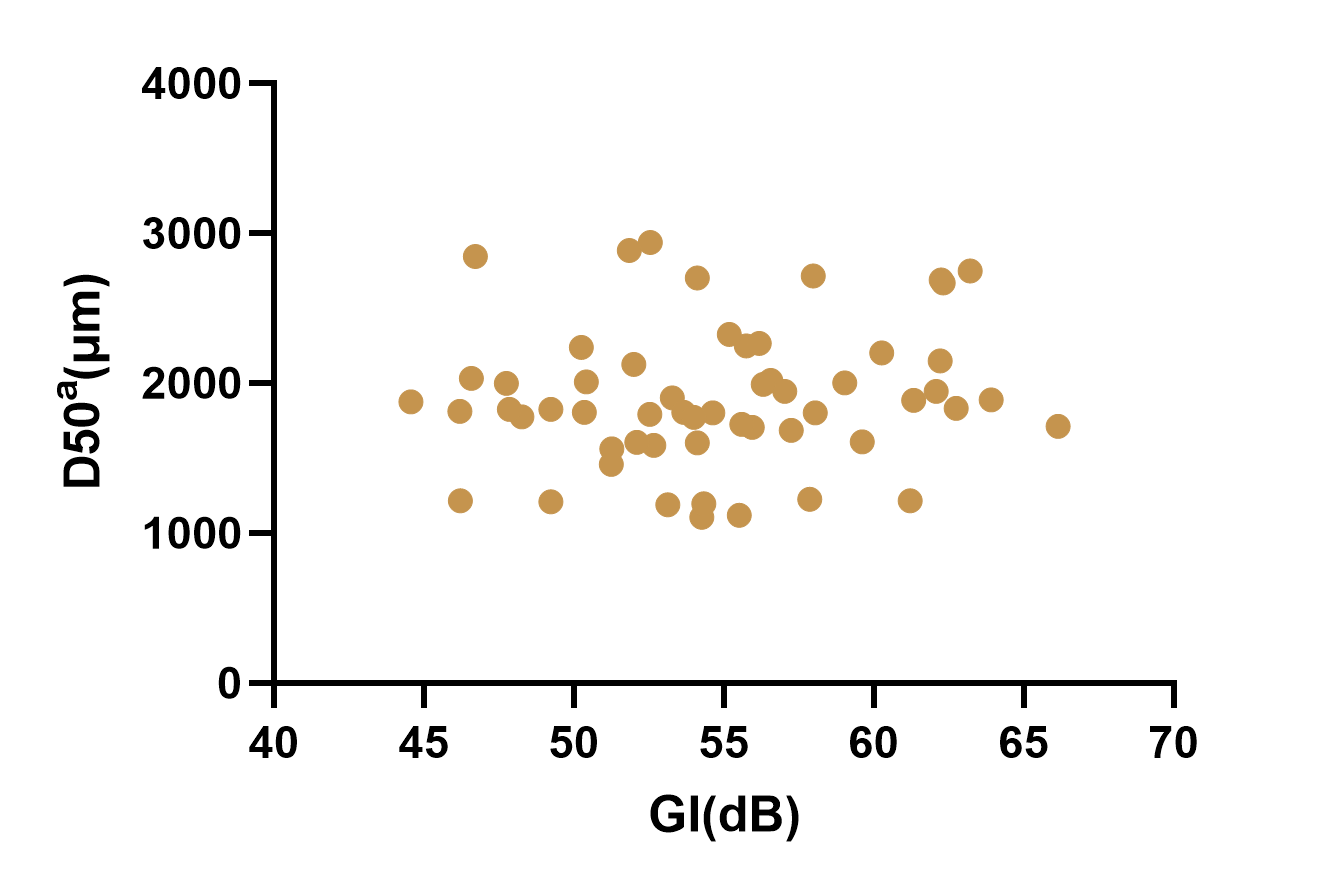

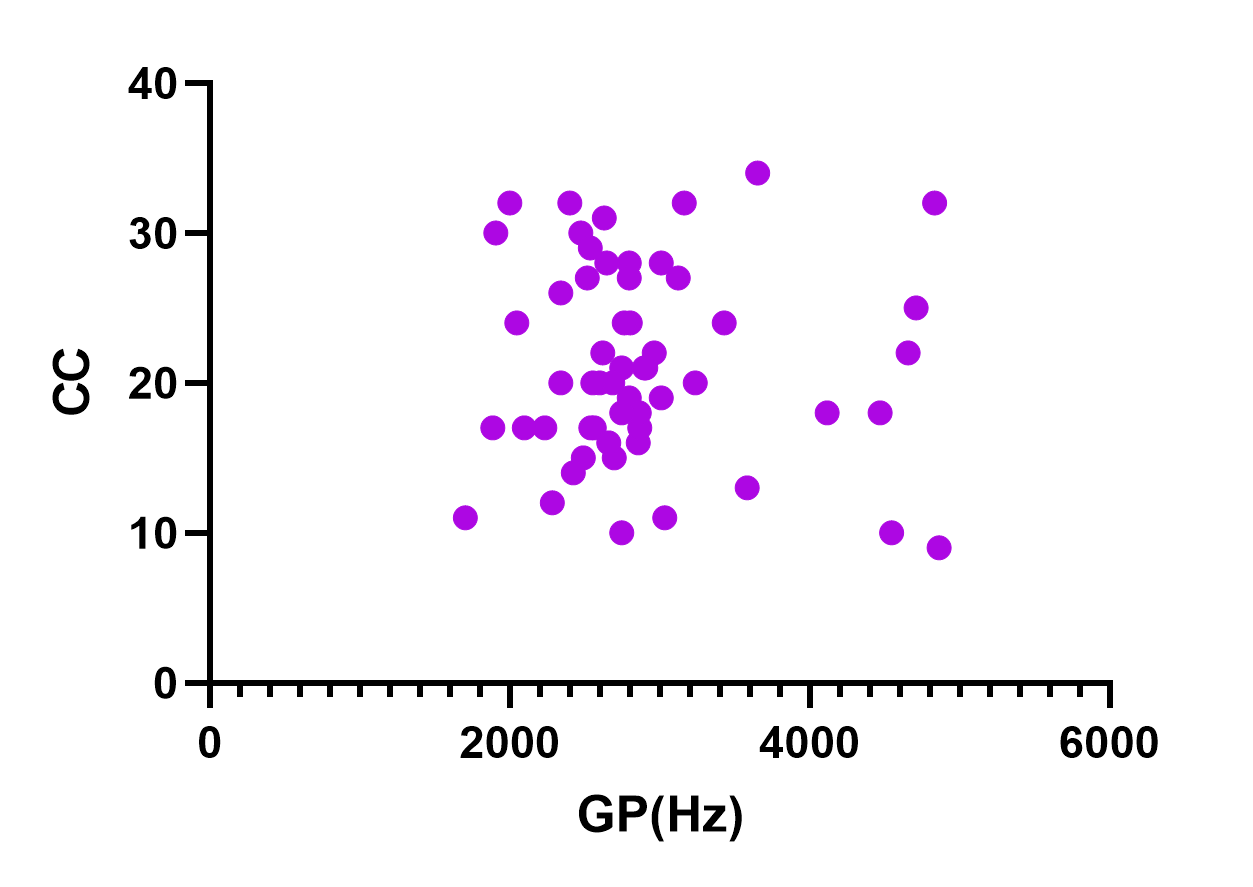

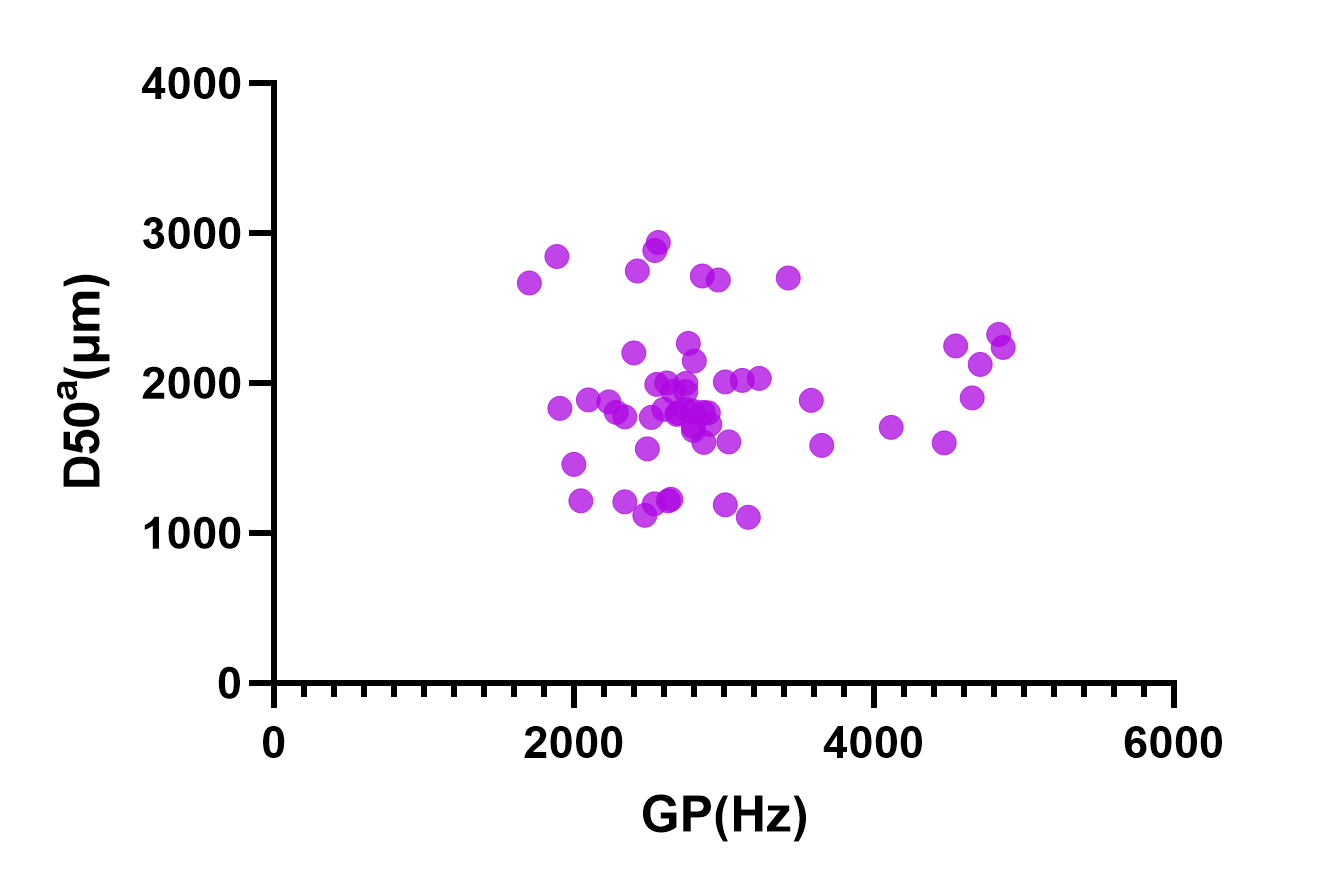

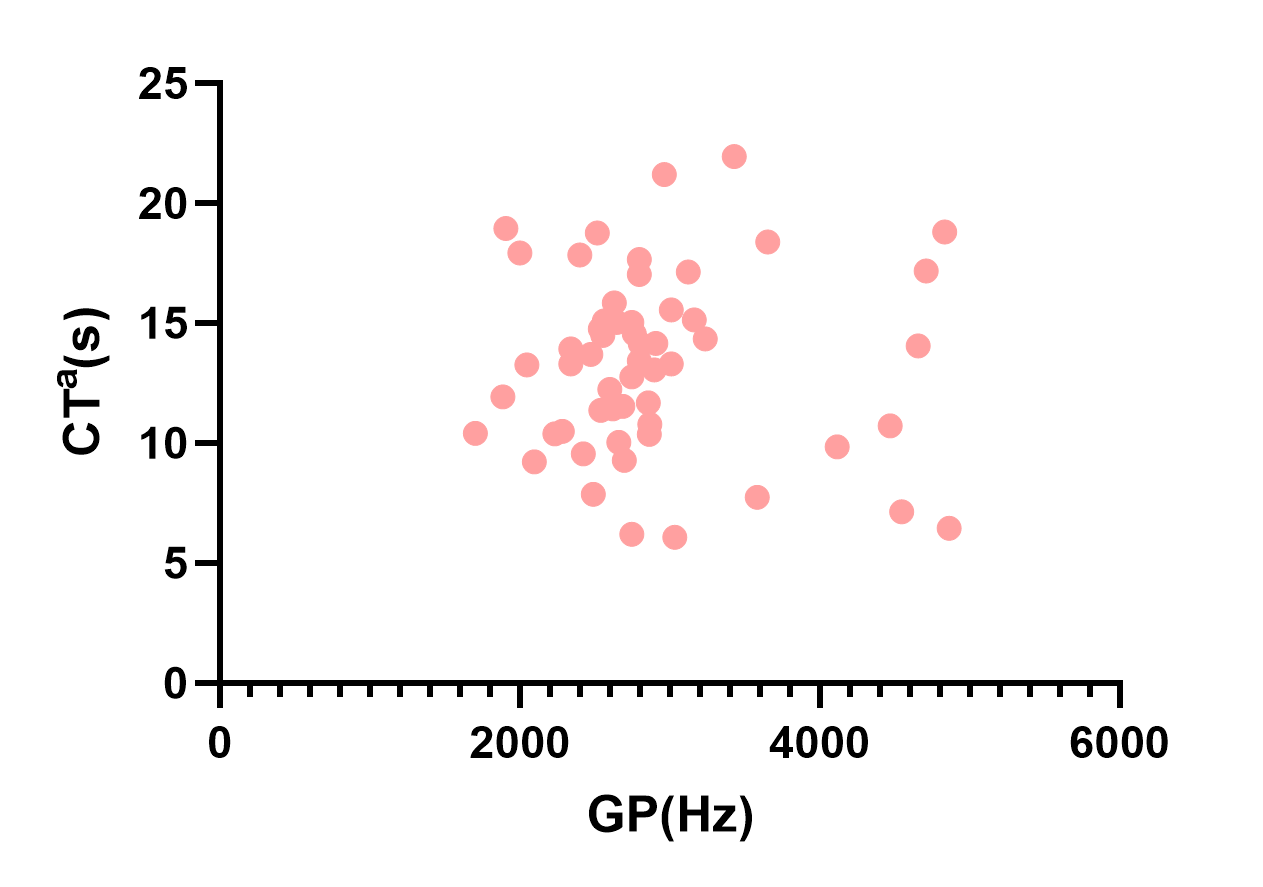

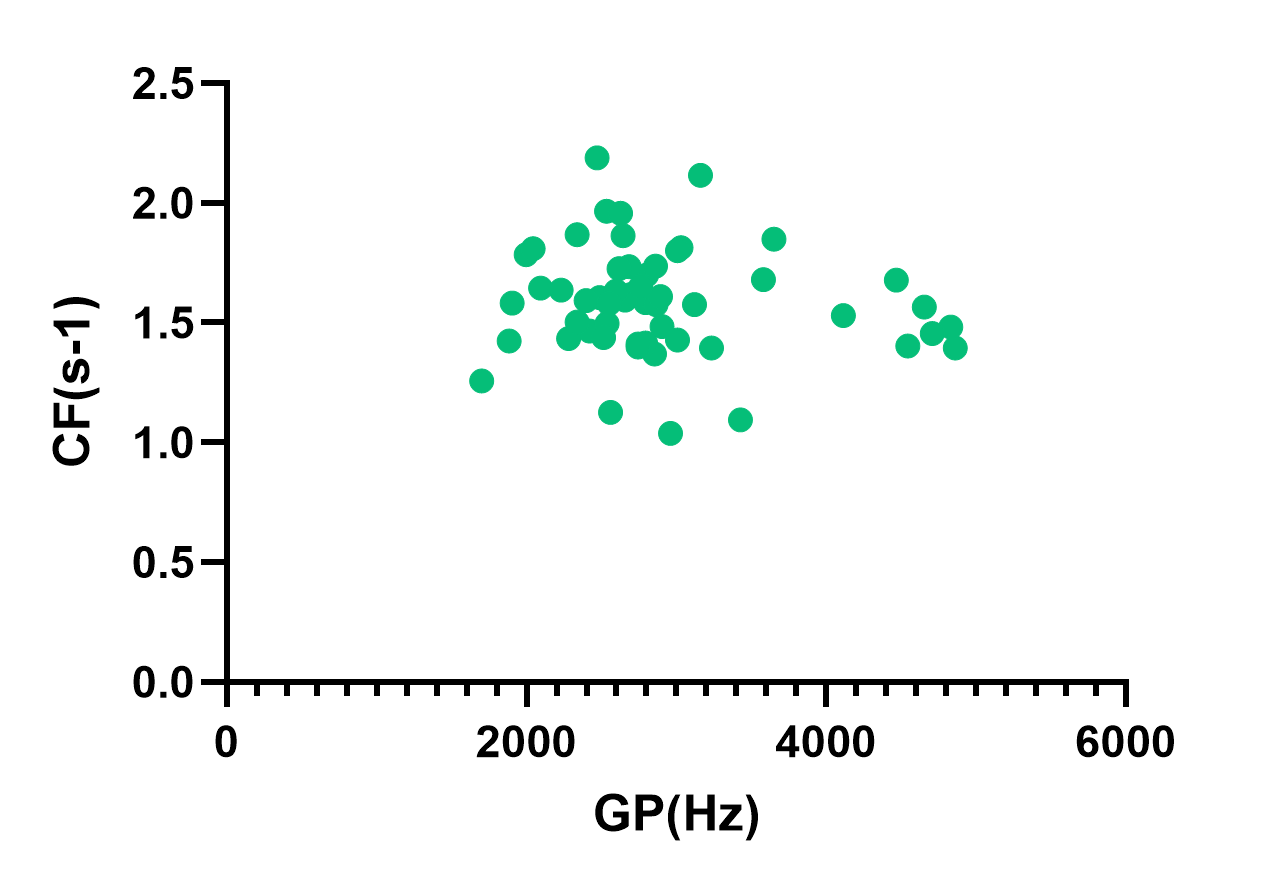

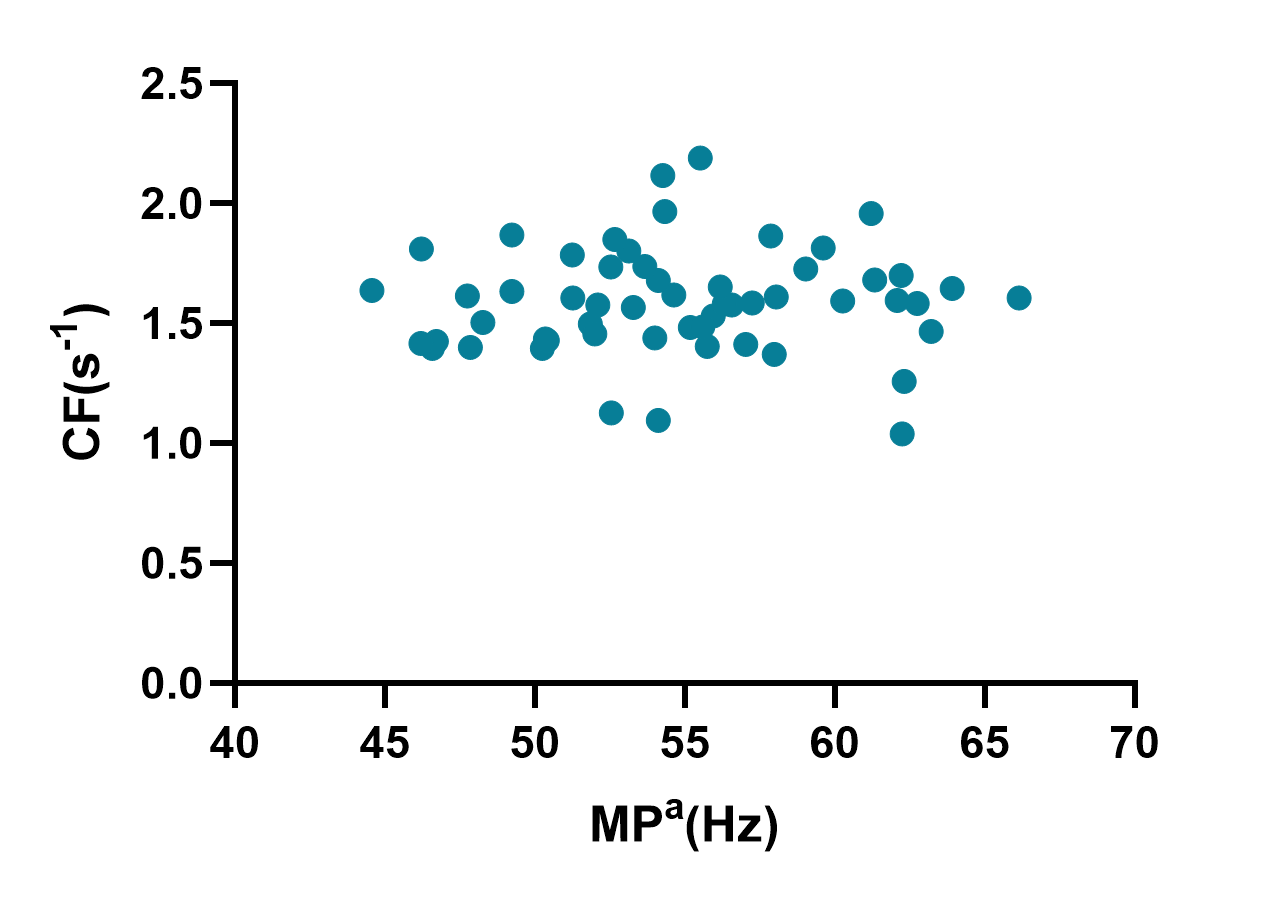

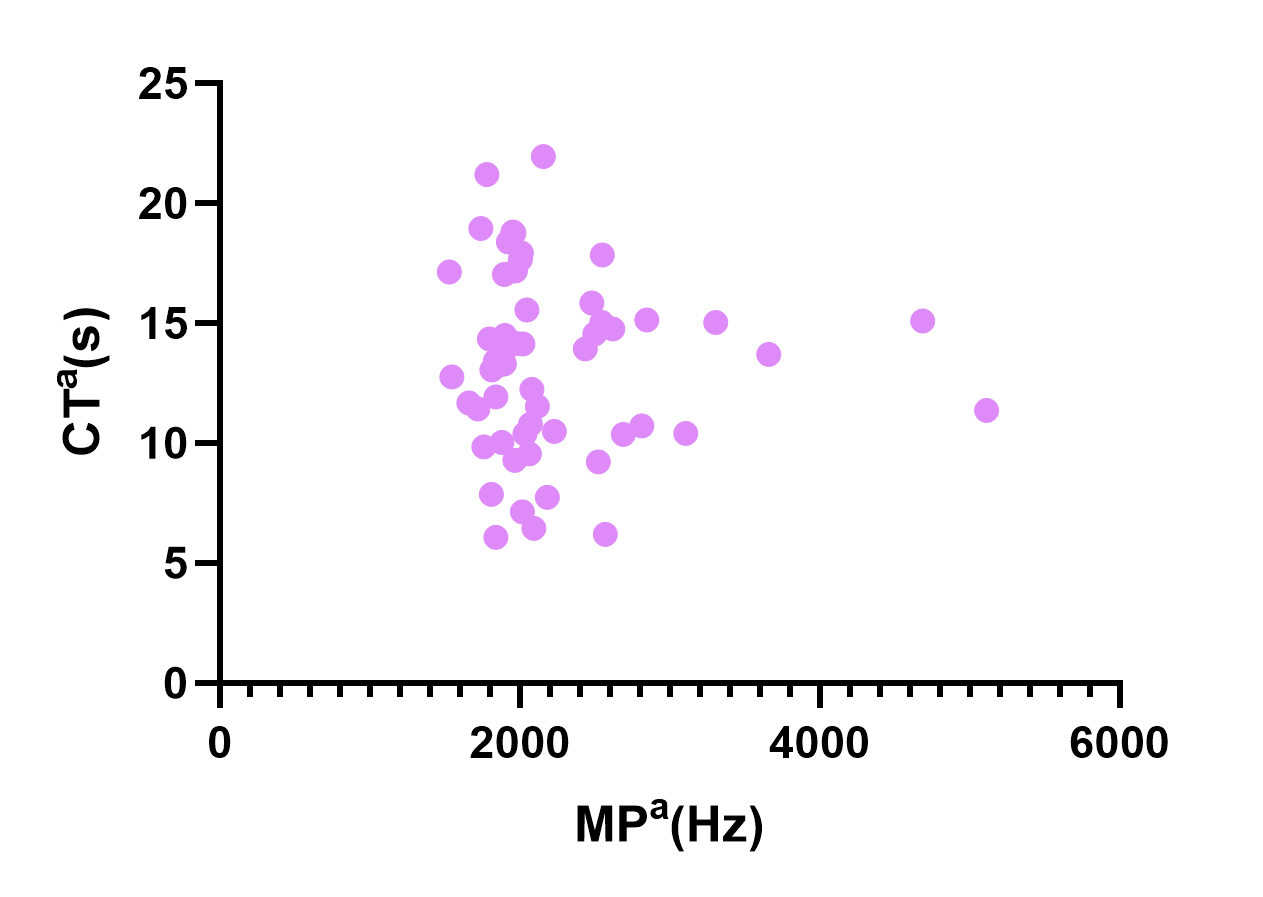

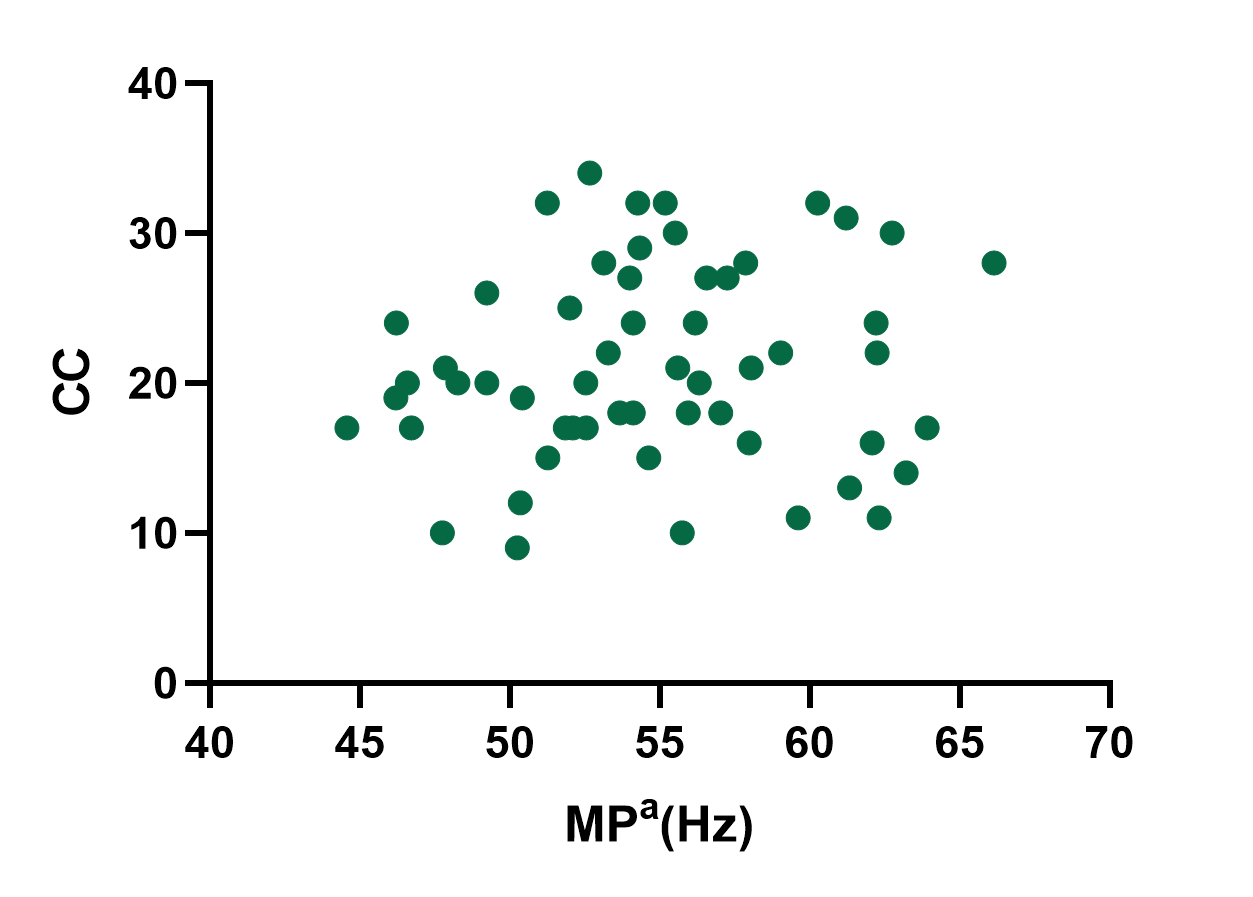

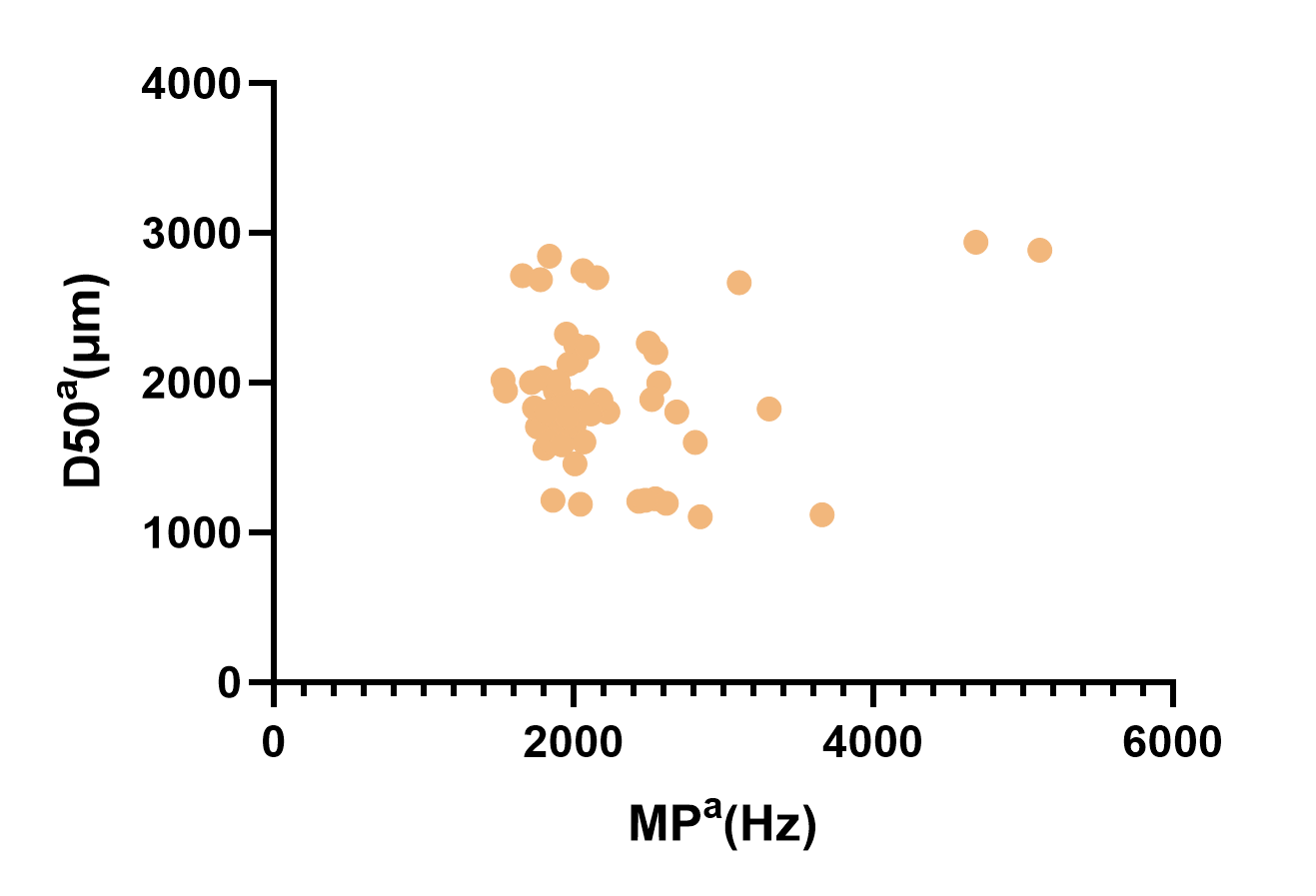

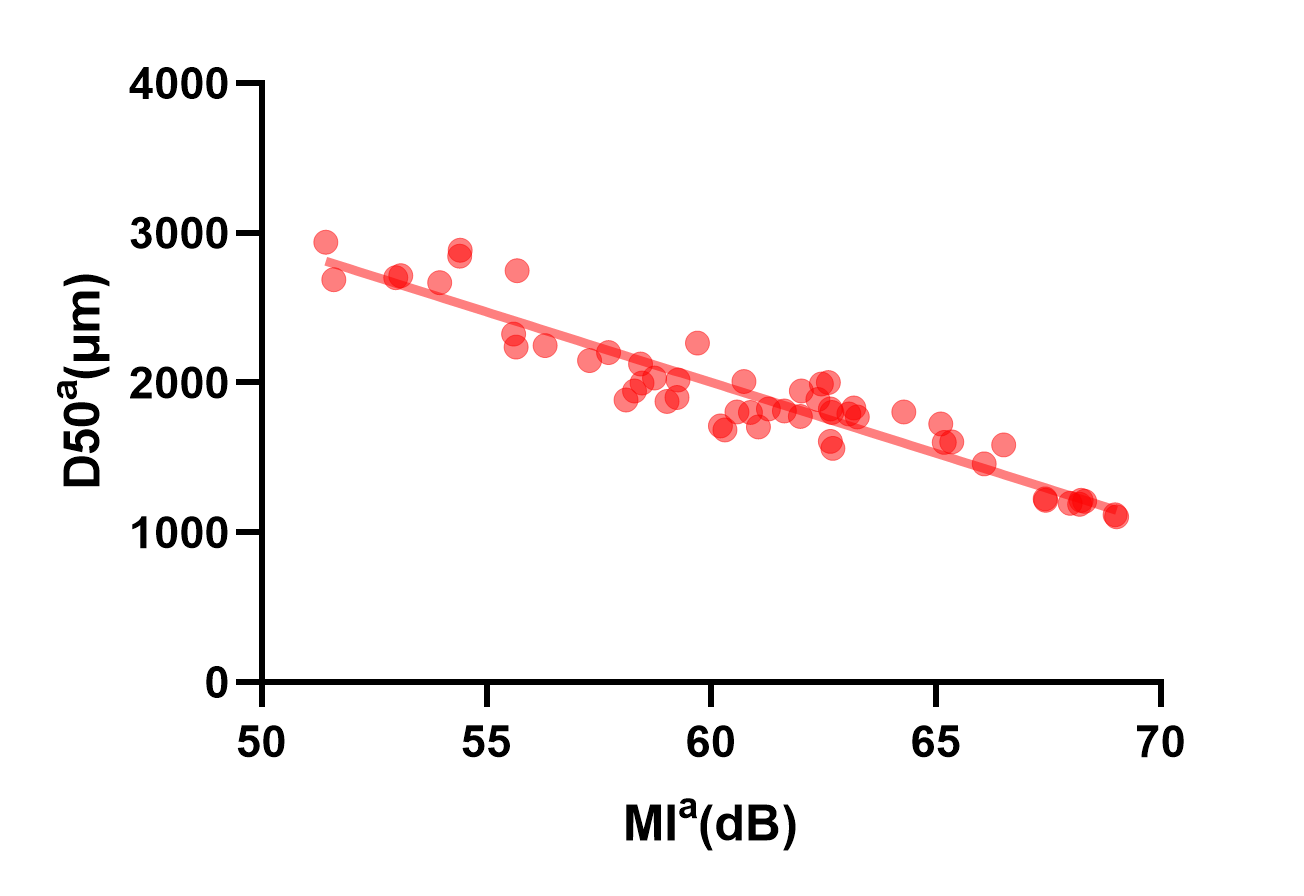

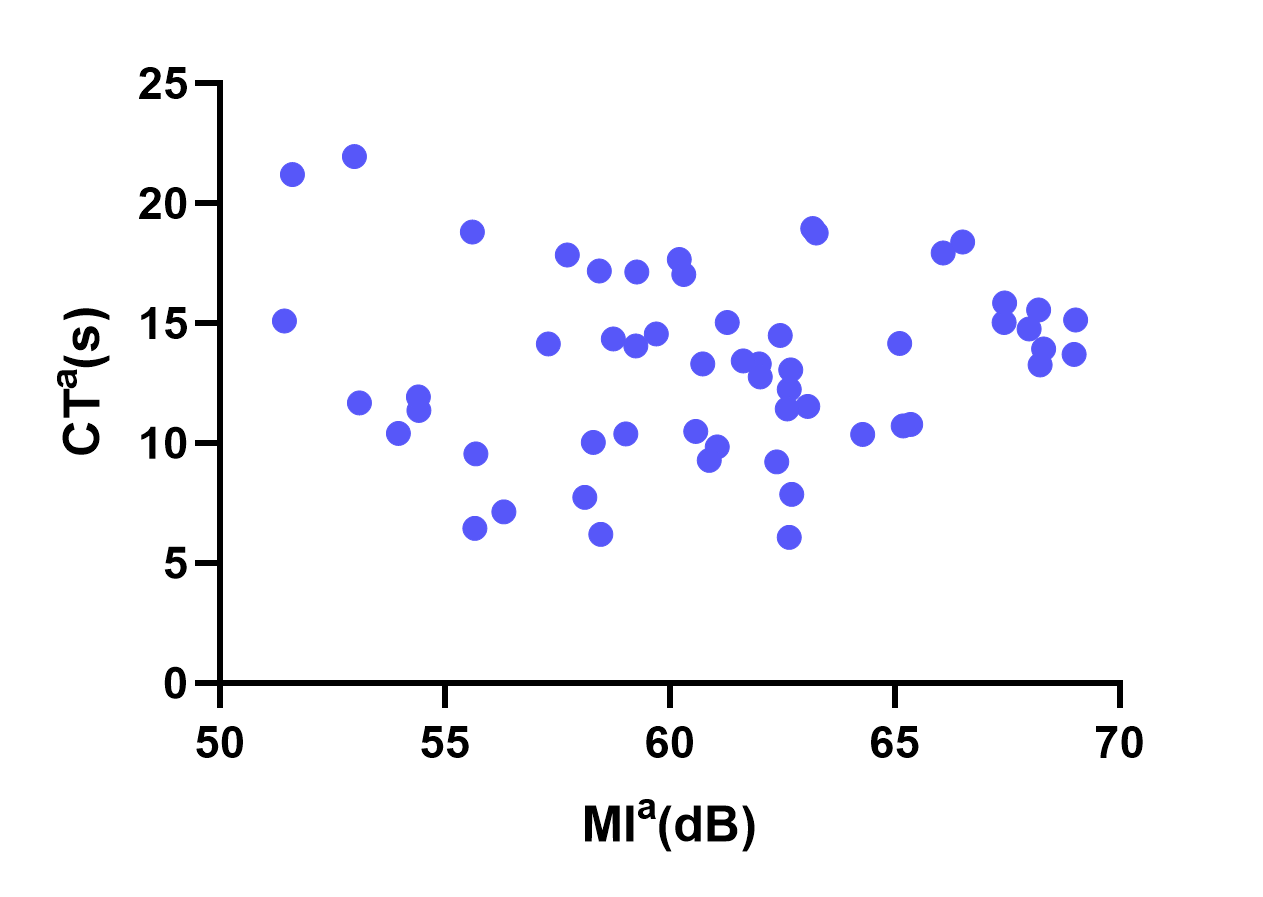

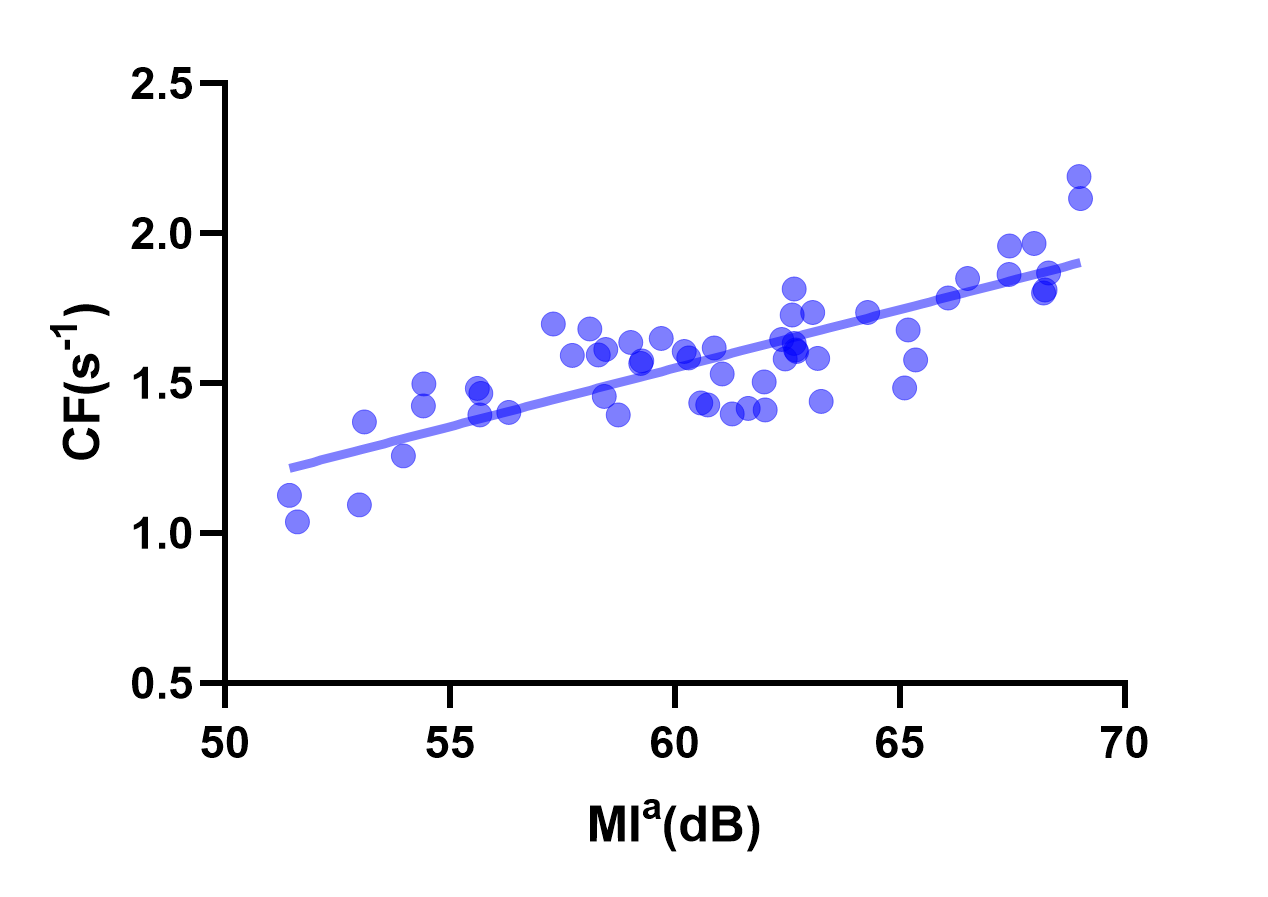

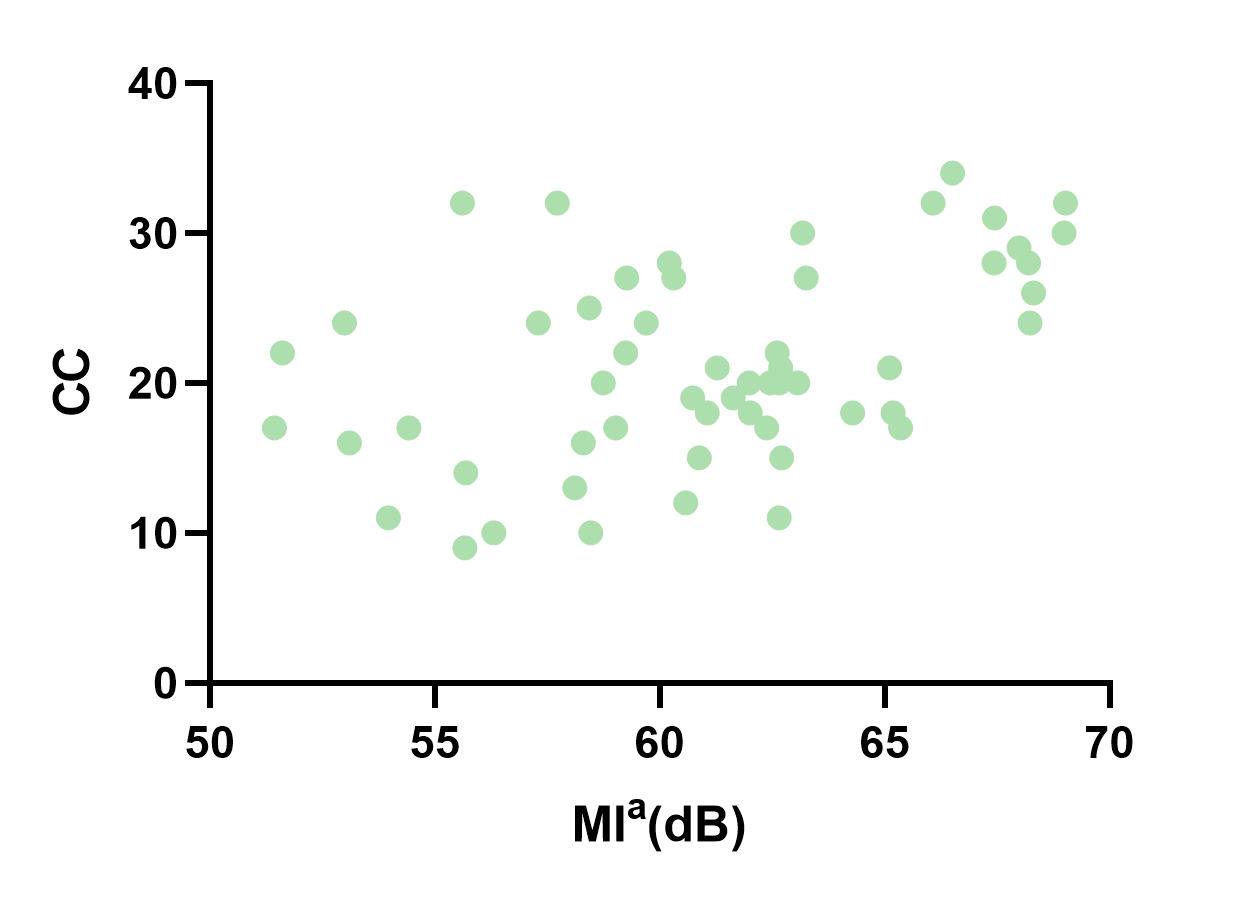
**

**Figure S1.** The scatter plots graph of the acoustic and masticatory parameters in the whole chewing sequence study.


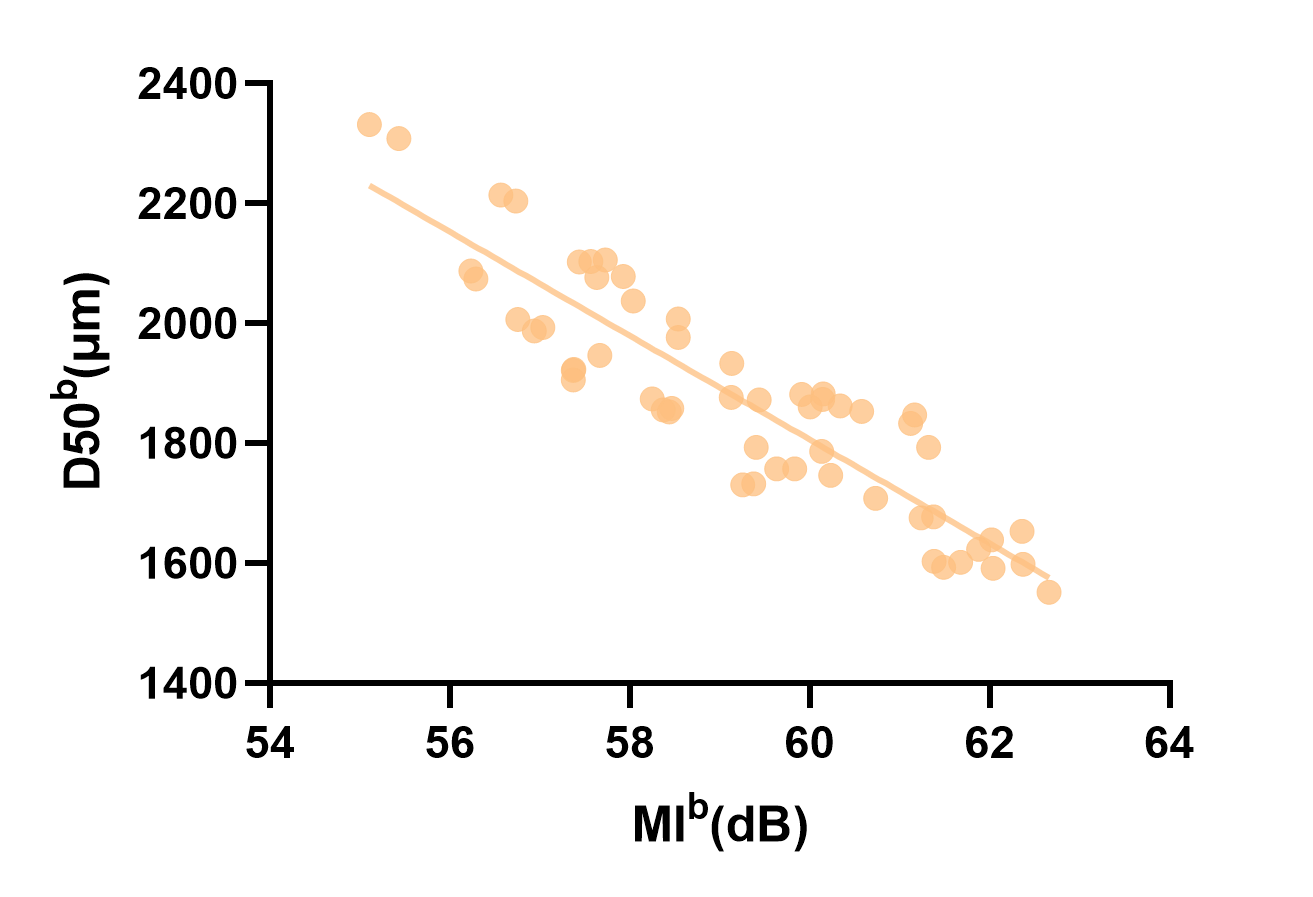

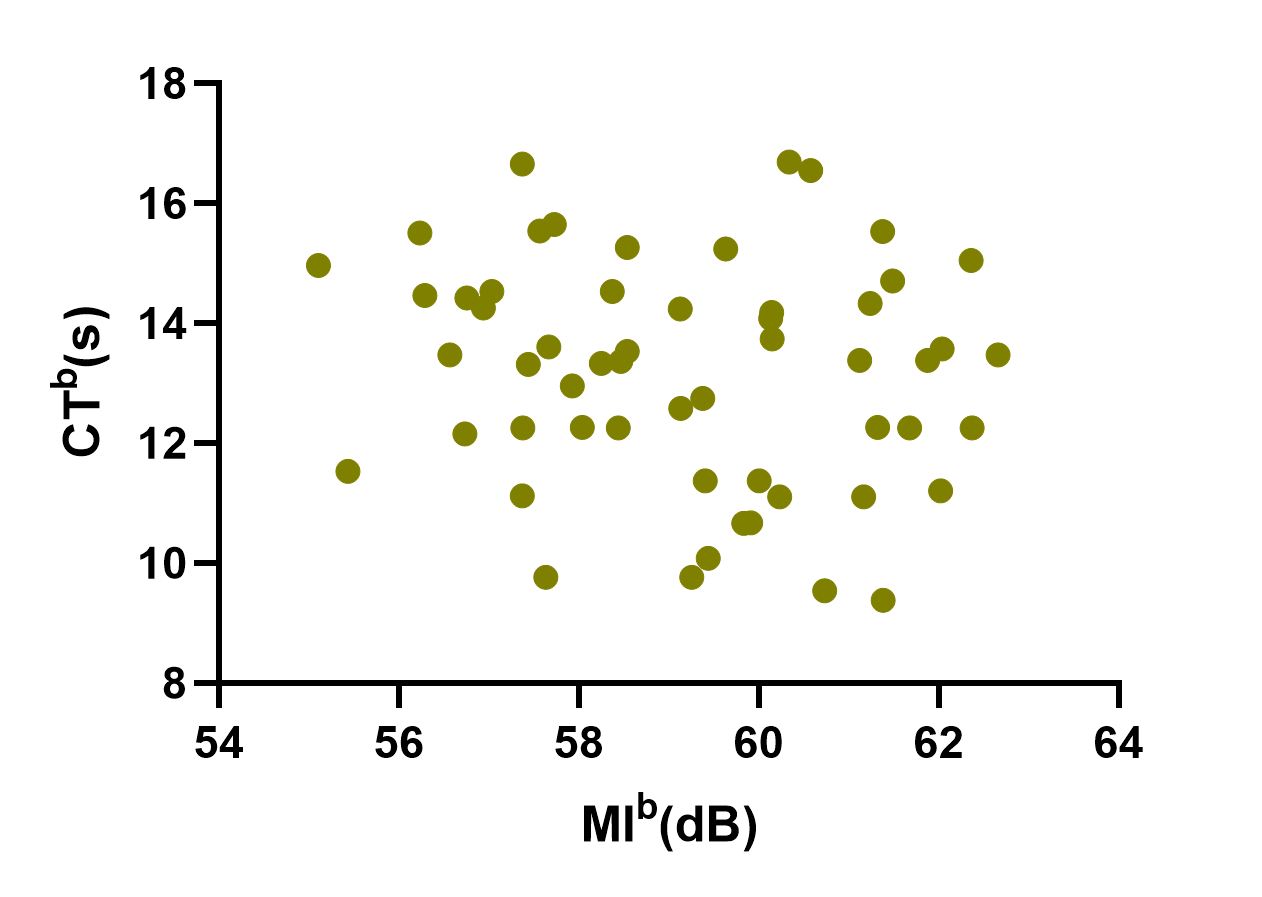

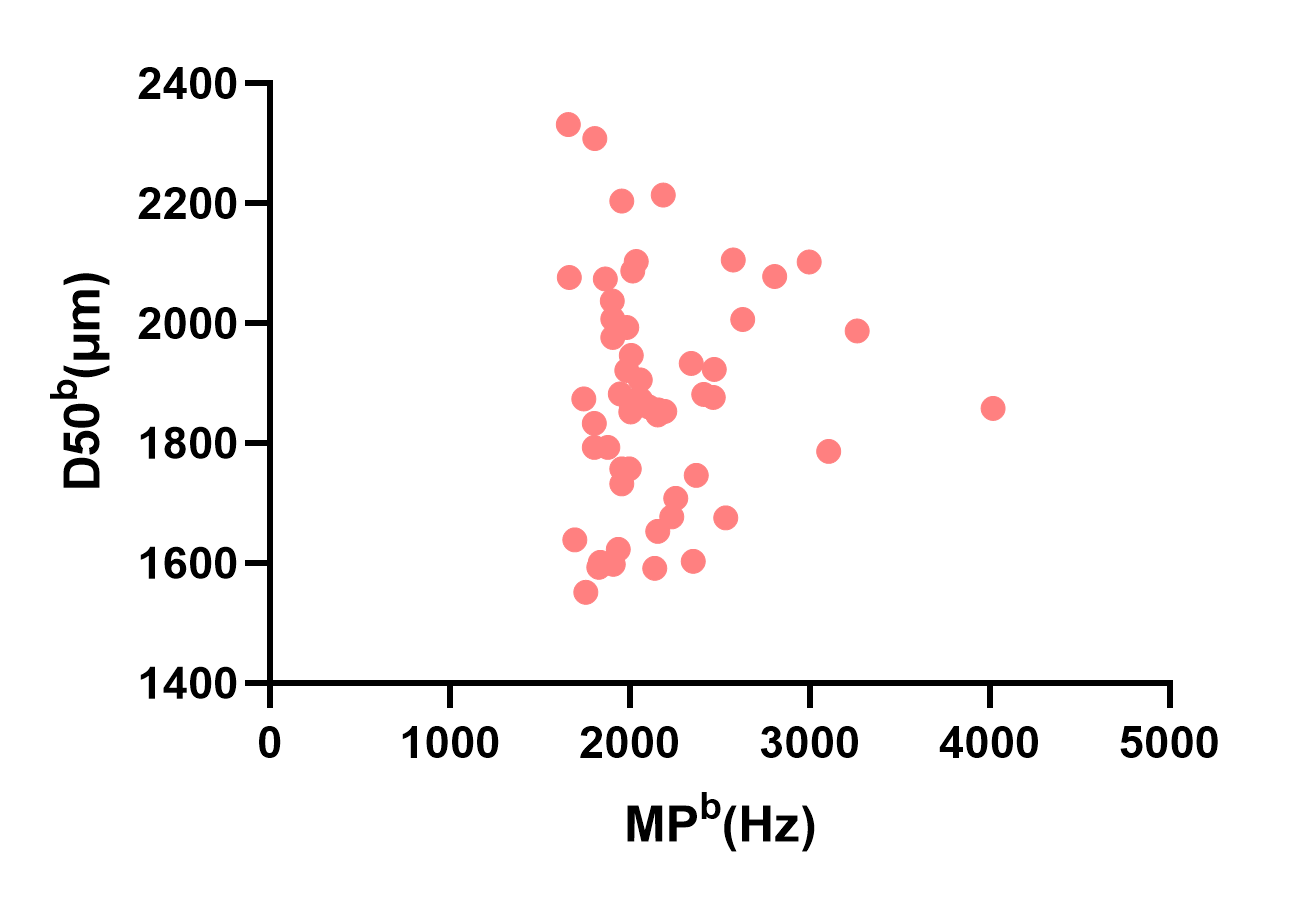

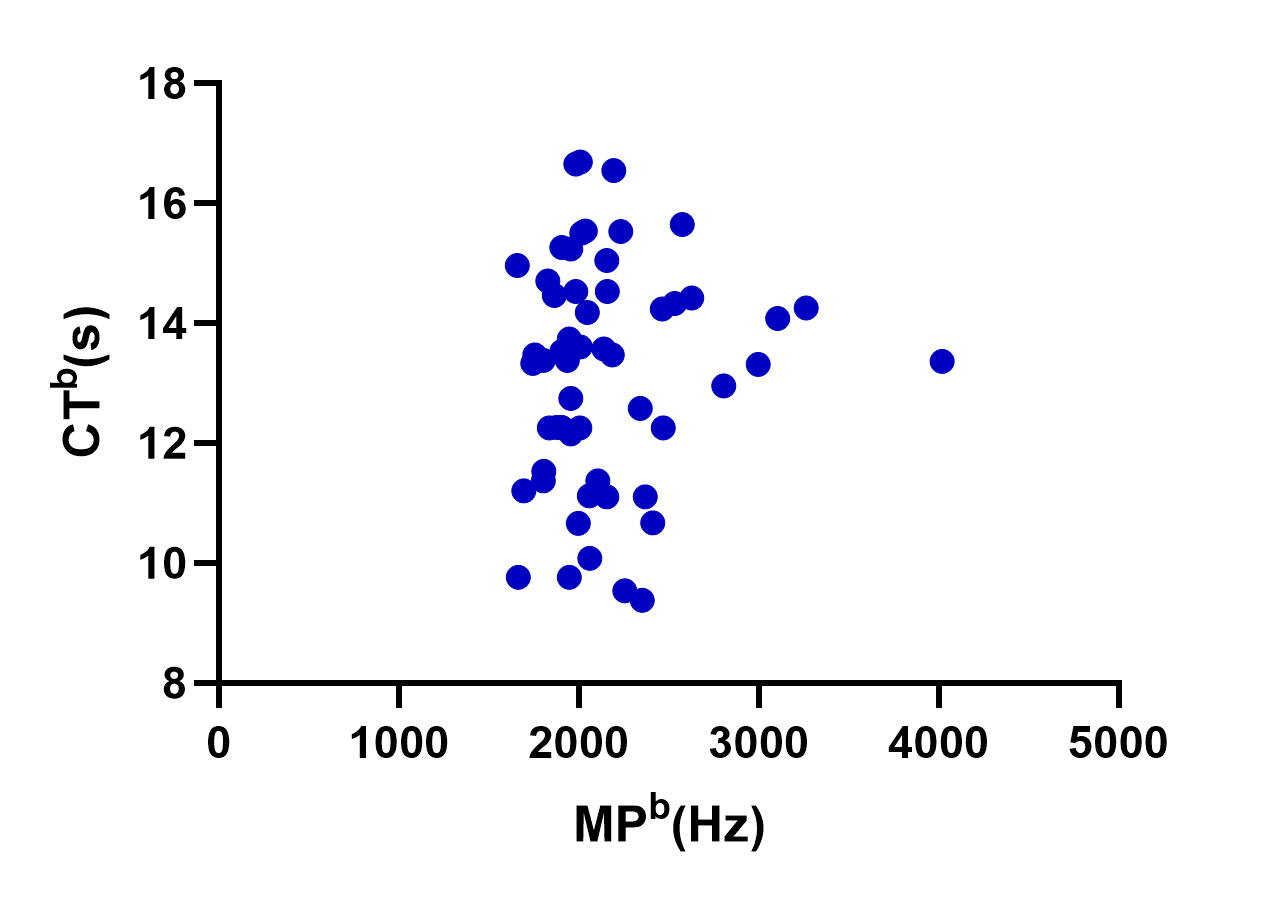


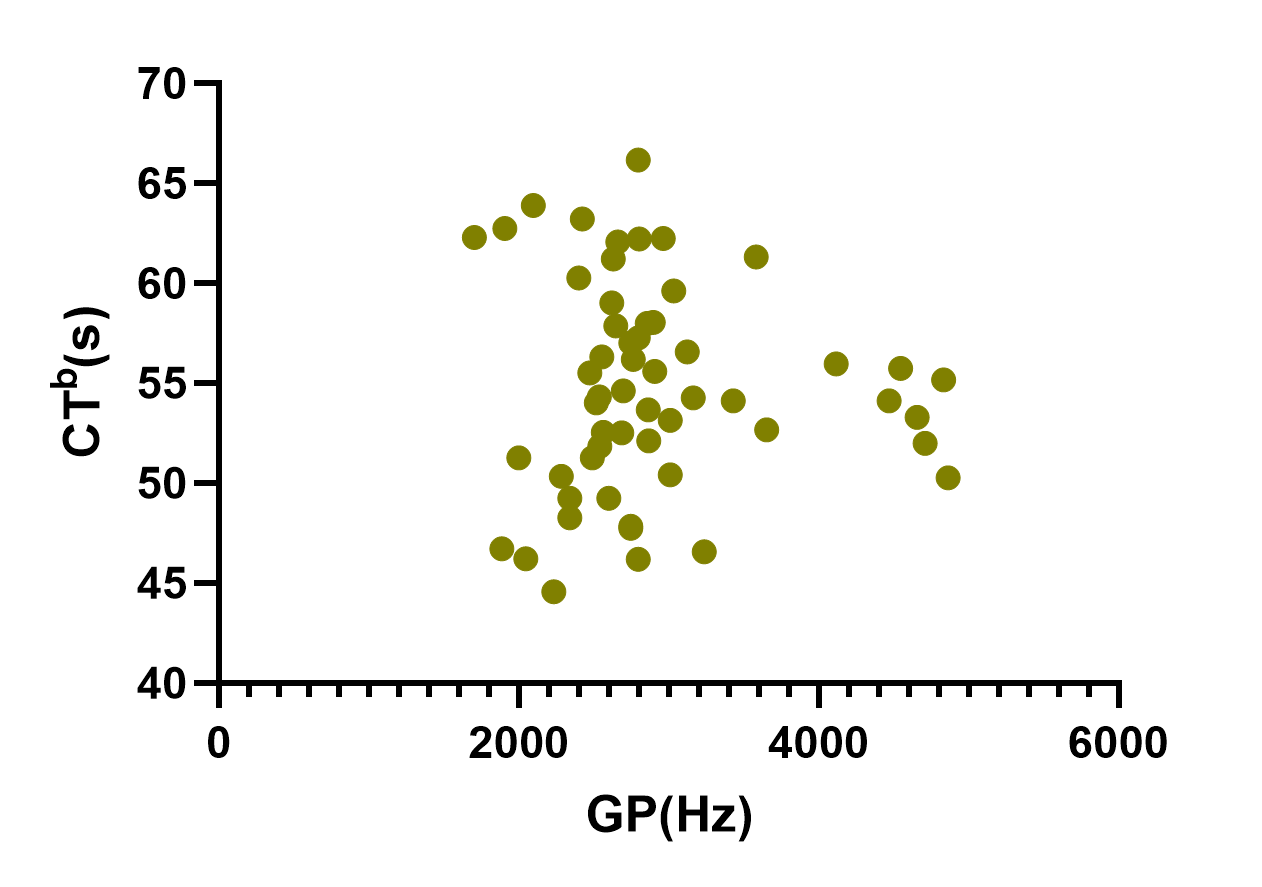

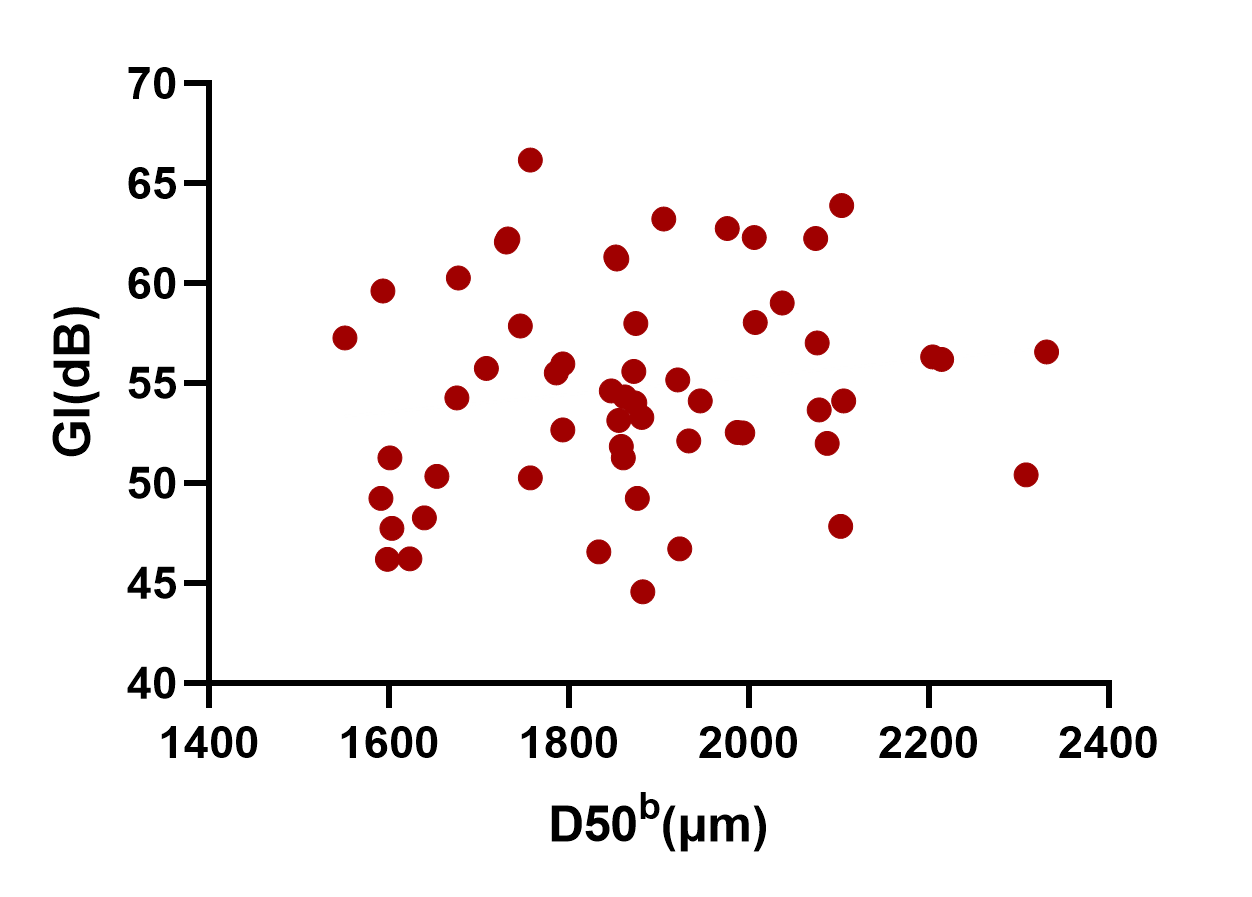

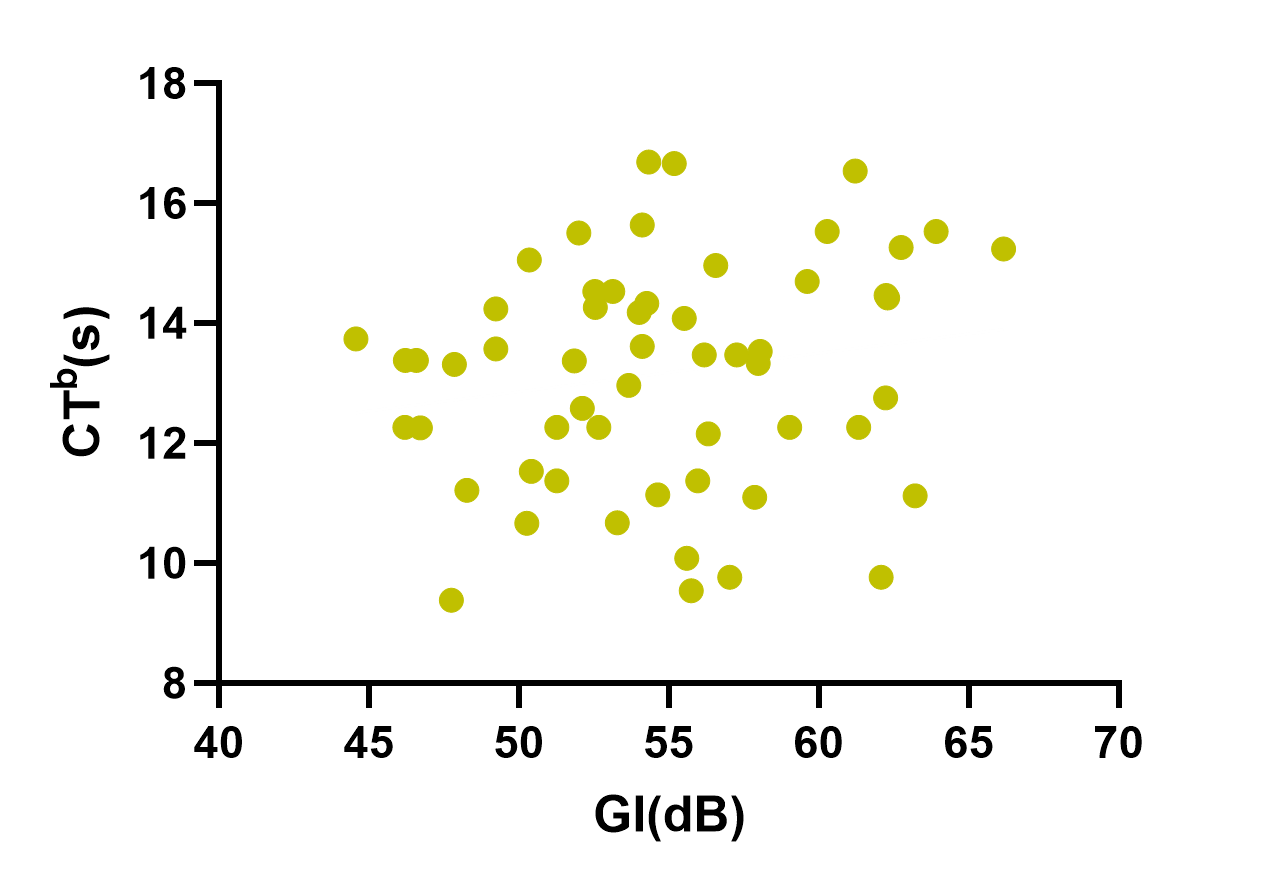

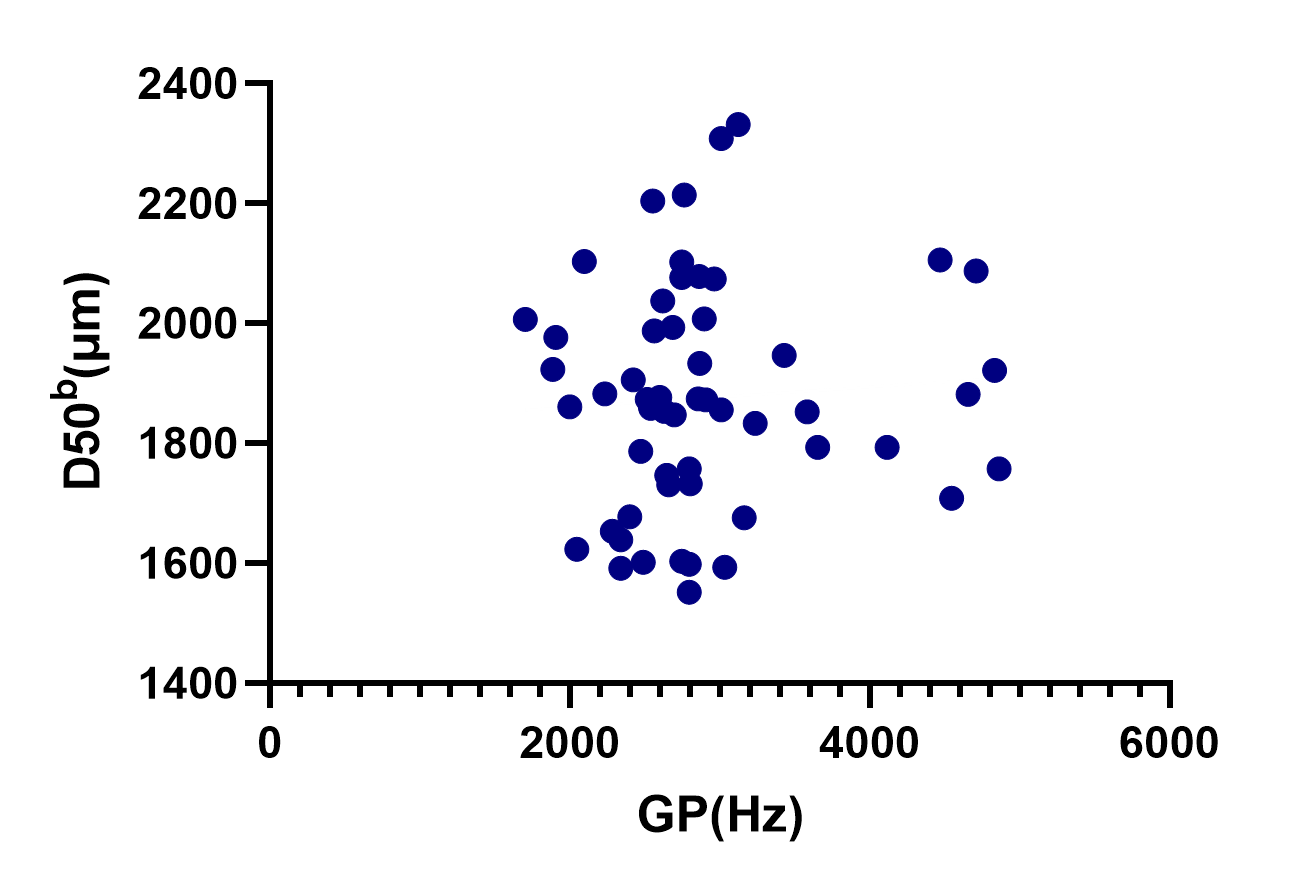


**Figure S2.** The scatter plots graph of MI^b^, MP^b^, D50^b^ and CT^b^ of in the fixed chewing strokes (21 times) study.

**
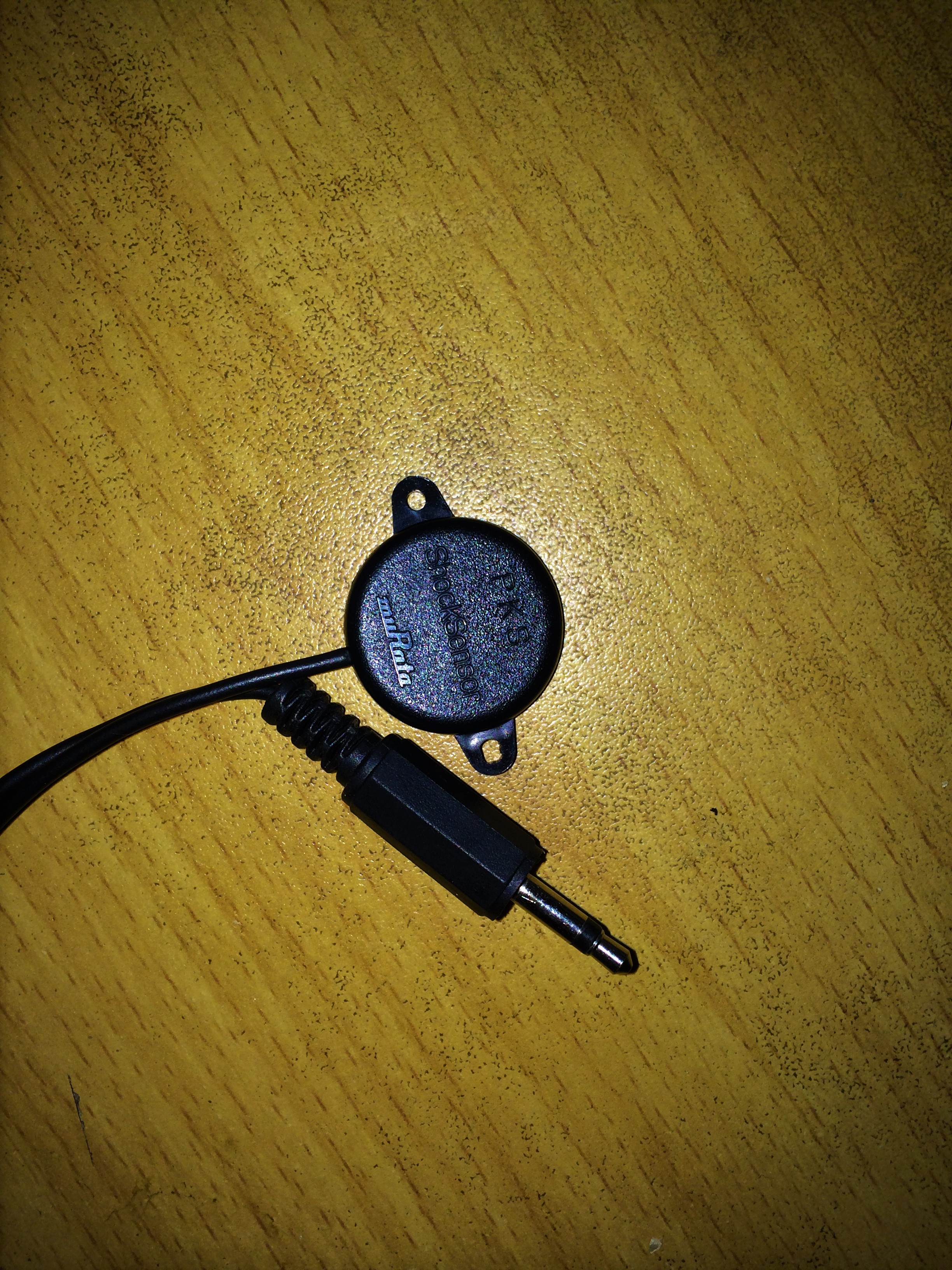

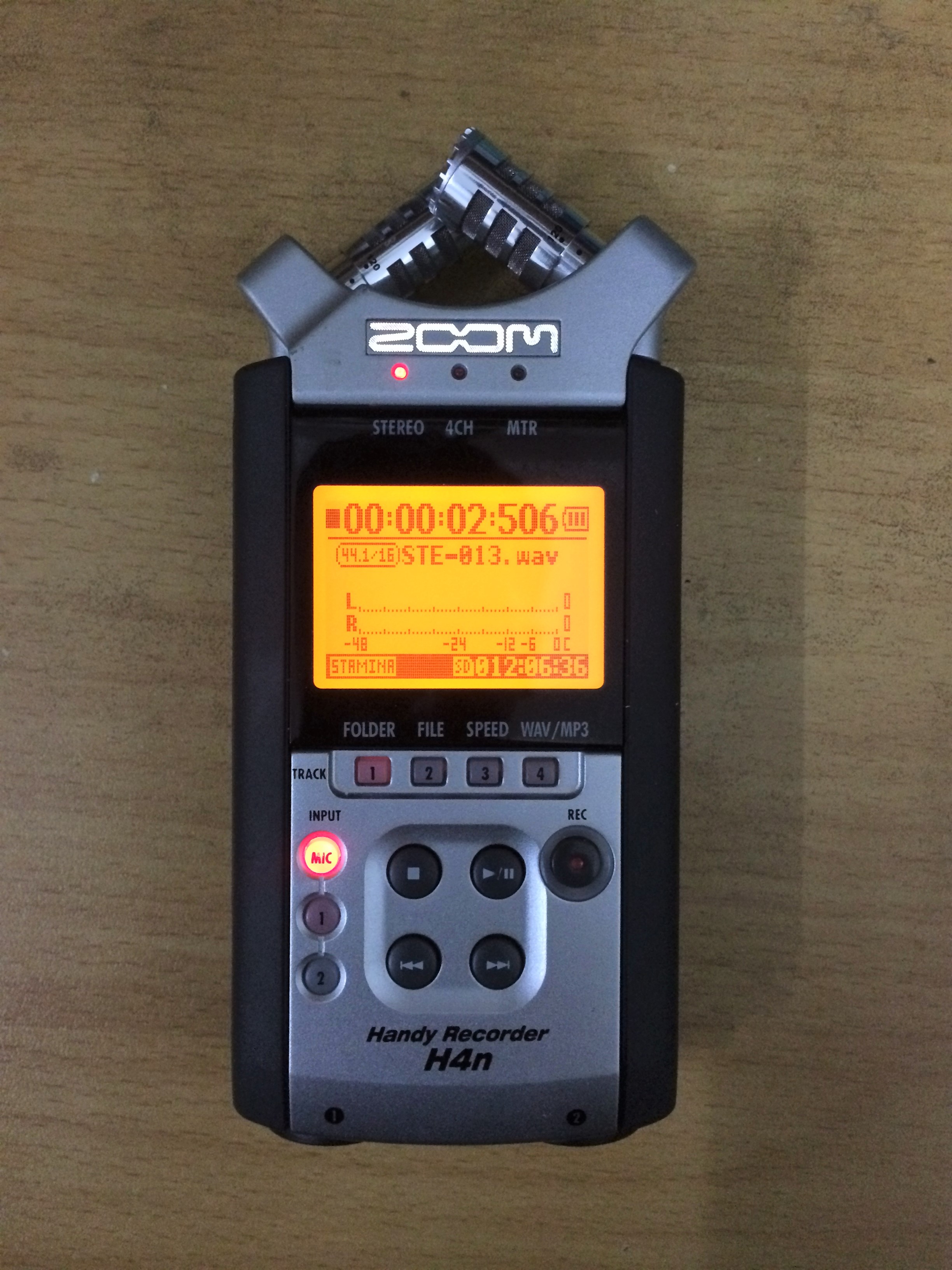
**

**
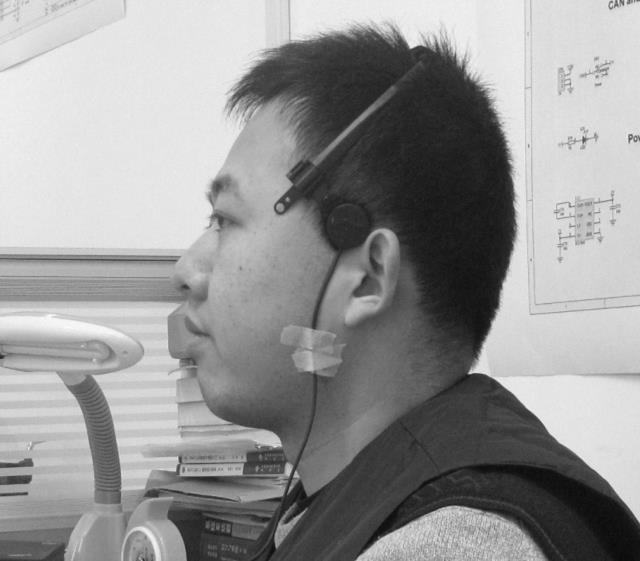
**

**Figure S3.** The bone-conduction microphone and Sony record device used in this study
